# Behavior change technique delivery during routine smoking cessation advice in primary care and associations with abstinence

**DOI:** 10.1093/tbm/ibag035

**Published:** 2026-07-03

**Authors:** Emma M Brown, Fabiana Lorencatto, Tim Coleman, A Toby Prevost, Stephen Sutton, Felix Naughton

**Affiliations:** Lancaster Medical School, Faculty of Health and Medicine, Lancaster University, Lancaster, United Kingdom; Centre for Behavior Change, University College London, London, United Kingdom; Centre for Academic Primary Care, School of Medicine, University of Nottingham, Nottingham, United Kingdom; Nightingale-Saunders Clinical Trials and Epidemiology Unit, King’s College London, London, United Kingdom; Department of Public Health and Primary Care, University of Cambridge, Cambridge, United Kingdom; School of Health Sciences, Faculty of Medical and Health Sciences, University of East Anglia, Norwich, United Kingdom

**Keywords:** smoking cessation, primary care, treatment guidelines, behavior change techniques, abstinence

## Abstract

**Background:**

Little is known about the content of routine smoking cessation support in primary care and how it relates to abstinence. While 121 behavior change techniques (BCTs) have been identified for cessation treatment, English treatment guidelines recommend 30 across cessation consultations.

**Purpose:**

We sought to identify: (1) BCTs delivered during routine cessation support in primary care in England; (2) associations between abstinence and (a) the total number of BCTs used, (b) delivery of the 30 recommended BCTs, (c) delivery of individual BCTs.

**Method(s):**

In an evaluation trial across 32 English General Practices, 149 pre-quit consultations (across 29 practitioners trained in cessation support) from a multisession cessation program were audio-recorded, transcribed, and analyzed for BCT delivery using a modified BCT taxonomy v1 and smoking-specific taxonomy. Abstinence was collected at 4-weeks (biochemically verified), 8-weeks (primary outcome, self-reported), and 6-months (self-reported) post quit date. Multilevel modelling assessed associations.

**Results:**

Of 121 BCTs, 85 were delivered in at least one consultation. A mean of 13.4 (SD = 2.76) of recommended BCTs were delivered, with 33% (10/30) delivered in at least half of the consultations. Total BCTs delivered were not associated with abstinence. Delivery of more guideline-recommended BCTs was only associated with abstinence at 4 weeks (*OR* = 1.03, 95% *CI* 1.00–1.06; *P* = .03). One BCT was positively associated, and one negatively associated with abstinence.

**Conclusions:**

One-third of recommended BCTs were delivered in at least 50% of initial consultations, with delivery associated with short-term abstinence. Limited practitioner time may constrain BCT delivery and is a consideration for future guidelines.

**Clinical Trials information:**

The Clinical Trials Registration #ISRCTN 56702353.

Implications **Practice:** Smoking cessation support can be implemented and can provide effective abstinence in primary care settings. Providing more behavior change techniques as recommended within treatment guidelines should be a focus, rather than the total number of all possible behavior change techniques.

**Policy:** The implementation of smoking cessation support that can provide effective abstinence in primary care settings may benefit from policy level changes with allocation to time, resources, and sufficient training.

**Research:** Future research should consider the nature of behavior change delivery in primary care (e.g. limited time, training) prior to the development and implementation of treatment guidelines for smoking cessation. It would be useful to consider specific treatment guidelines for primary care practitioners.

## Background

Smoking remains the single largest cause of preventative premature morbidity and mortality worldwide [1]. Approximately 1.1 billion people smoke, which is estimated to cause more than 7 million deaths per year [2]. In the United Kingdom alone, 115,000 deaths per year are attributable to smoking with 54,300 deaths from cancer [3] including lung cancer, which has one of the lowest cancer survival rates [4]. Specialist stop smoking programs delivered in the community by specialist stop smoking practitioners play a significant role in helping smokers to quit and are accessible in 23 countries worldwide [1]. The English stop smoking services specifically help smokers to quit by providing evidence-based behavior change strategies or techniques (BCTs) such as goal setting and action planning. BCTs are informed by behavior change theory [5], alongside smoking cessation medications [6] and are recommended by the National Centre for Smoking Cessation and Training (NCSCT) [7]. In order to maximize the impact of these services, there is a need to identify and promote best practice relating to the advice and support delivered to smokers.

Primary care is the second largest setting where cessation support is delivered in England [8]. While effective [9], quit success rates in primary care are among the lowest of all settings, with 43% of clients seeking out this support successfully quitting by 4-week follow up [8]. Stop smoking practitioners in primary care (i.e., primary care nurses or healthcare assistants) are expected to deliver most cessation support during an initial consultation, lasting approximately 15 min. This routine support guided by the NCSCT includes the delivery of BCTs relating to: the assessment of current readiness and ability to quit, explaining the purpose of and conducting carbon monoxide monitoring, facilitating barrier identification and problem solving, facilitating goal setting (i.e., a quit date), providing information on withdrawal symptoms, advising on a stop smoking aid (including a prescription), and arranging a follow-up appointment [7, 10].

Evidence to date indicates that the content of support delivered by stop smoking practitioners varies substantially [11]. Primary care practitioners provide cessation advice as only one part of a typically varied healthcare role and receive less training and supervision. Primary care practitioners are, therefore, anticipated to deliver cessation support that includes fewer and a more varied range of BCTs than recommended in guidance. This likely variation in support delivery may in turn be related to success rates, but this is currently unknown.

There is a need to identify what cessation support is routinely delivered in primary care to identify opportunities to improve effectiveness [8, 12]. Furthermore, while some BCTs have been associated with 4-week smoking abstinence at the service level [13], there is currently no evidence at the individual level, for any type of practitioner, as to whether specific BCTs are associated with abstinence outcomes. This is an important distinction as smoking cessation occurs at the individual level, whereby service level findings may reflect service characteristics rather than individual effects.

Therefore, the aim of this study was to apply behavior change technique (BCT) taxonomies [14–16] to identify (1) the BCTs delivered to individuals during routine cessation support in primary care, and consistency with treatment guidelines, (2) the association between abstinence and (a) the total number of BCTs delivered, (b) BCTs delivered as recommended by treatment guidelines, and (c) the delivery of individual BCTs (exploratory analysis).

## Method

### Design

This study uses data from the iQuit In Practice (iQIP) trial [17] as an observational cohort. The iQIP trial was not intended to alter routine cessation support delivered by primary care practitioners but evaluated an adjunctive tailored cessation advice report and a 3-month text message cessation program. For this current study, there was a focus on the cessation support delivered in the initial consultation, which was a standalone stop smoking appointment, as in primary care the majority of support is delivered during the initial consultation [7, 11] and attendance at follow-up consultations in this setting is low. For example, 74% of the iQIP participants (smokers) attended at least one follow-up, but only 29% attended two or more follow-up consultations [17]. While NCSCT standard treatment guidelines were available for all practitioners delivering stop smoking support, they are not specific to the primary care setting.

### Participants

Fifty-three primary care practitioners (primary care nurses or healthcare assistants) delivering stop smoking usual care to trial participants (smokers) were asked to audio-record a purposively sampled subset of their initial intervention consultations (the 3rd, 4th, 5th, 6th, 7th, 8th, 19th, and 20th initial participant [smoker] consultations)—this order enabled practitioner’s to “warm up” before starting to record consultations, and to minimize complexity while enabling a sample across patients. This included recording the usual care consultation and completion of an online questionnaire with the participant (smoker), which was part of iQIP trial procedures and used for tailoring iQIP support [17]. The questionnaire recordings were not included in this study. In total, 159 audio-recordings were collected across 29 practitioners (54.7%), though 10 recordings were removed from analysis due to being incomplete or inaudible. This left 149 recordings for analysis, representing 149 participants (smokers). Of those practitioners not providing a recording, eight (15.1%) recruited fewer than three participants (smokers) in the host trial (the recording threshold), leaving the remaining with an unknown reason for not recording, though nine of these (17.0%) only recruited three to five participants (smokers), reflecting intermittent recruitment or engagement. All practitioners involved had received training, as part of their role, to deliver standard stop smoking support to a standard known as “level 2” advice [11].

Of the 29 practitioners who made audio-recordings, 25 recorded three or more stop smoking consultations, which was considered sufficient to estimate the typical BCT delivery of that practitioner, based on evidence of relatively stable within-practitioner cessation support delivery [11]. All participants (smokers) in the trial seen by these 25 practitioners (*n*= 459) were included in the exploratory analysis addressing objective 2c, by using a calculated “delivery score” if no recording had been made (see Data analysis section). Ethical approval for the trial, including the audio-recorded consultations, was received by Cambridge Central NHS Research Ethics Committee (reference: 09/H030/87).

### Procedure and materials

A coding tool was developed (Supplementary Material S1) using the smoking-specific BCT taxonomy [14] alongside a modified version (addition of *4.5 Tell to act*, and removal of *8.5 Overcorrection* and *12.5 Body changes*) of the cross-behavior generic BCT taxonomy v1 [15], which was used for the IC-Smoke Study [16], and with the addition of two BCTs recommended by our patient and public involvement group (*schedule follow-up appointment*, *physiological effects of nicotine/smoking*). BCTs that had some overlap with others between the taxonomies used were omitted (smoking-specific BCTT = 14) alongside those deemed irrelevant for smoking cessation by previous authors (modified BCTTv1/IC-Smoke = 2), leaving a total of 121 BCTs within the coding tool. Descriptions of BCTs in the context of smoking cessation consultations were included in the coding tool to enhance efficiency and maximize inter-coder reliability. Once audio-recordings were transcribed verbatim, the coding tool was used to analyze the practitioner’s speech in the session, to identify and categorize BCTs delivered. For example, “so, we’re going to put a quit date down as the fourth, okay?” was coded as BCT *Goal setting*. We used the NCSCT treatment guidelines (www.ncsct.co.uk), which recommend the use of 30 BCTs across the pre-quit and quit date consultations [7], to determine the recommended BCTs for delivery by primary care practitioners. While 30 BCTs were identified within the treatment guidelines across pre-quit (*N* = 24) and quit date (*N* = 22) consultations, many of these are repeated across both consultations (*N* = 16). The main difference between BCTs recommended at pre-quit and quit date is that BCTs are used to assess and comfort clients within a pre-quit consultation, and used to confirm and prepare in a quit date consultation. Given that primary care practitioners are expected to deliver the majority of cessation support during the initial consultation [7, 11], guideline recommend BCT’s from pre-quit and quit date consultations were evident and coded. Examples of transcribed BCTs can be found in supplementary Material S3.

### Measures

All participants (smokers) were asked to complete study follow up measures at 8 weeks, and 6 months post quit date, which included self-reported smoking outcomes. Smoking outcome data at 4-week follow-up was collected in practice by primary care nurses or healthcare assistants and validated using exhaled air carbon monoxide monitor readings using a Bedfont piCO Smokerlyzer. Smoking outcome data at 8-week and 6-month follow-up time points were self-reported and collected via online or telephone-based questionnaire. As this study was focused on short-term efficacy, the primary outcome was self-reported 2-week point prevalence at 8-week follow-up. Secondary outcomes were defined as carbon monoxide validated 2-week point prevalence at 4-week follow-up and self-reported 6-month continuous abstinence at the 6-month timepoint.

We also used data collected routinely at baseline as covariates in the analyses, including ethnic group, occupation code, daily cigarettes smoked and time to first cigarette on waking, and smoking cessation medication use.

### Data analysis

The mean length and standard deviations of all audio-recordings, and the mean duration of advice delivery were assessed using descriptive statistics.

To examine whether BCTs could be reliably coded using the developed coding tool, 15 (10% of the data) randomly selected transcripts were coded independently by the first author [E.M.B.] and second-coded by last author [F.N.]. BCT coding was assessed for inter-rater reliability using Cohen’s Kappa and percentage agreement using denominators of (a) all 121 BCTs and (b) only BCTs that had been coded at least once. Results of the inter-rater reliability were interpreted using Altman’s guidelines of <0.20 poor, 0.21–0.40 fair, 0.41–0.60 moderate, 0.61–0.80 good, and 0.81–1.00 very good reliability [18]. Once reliability was demonstrated, the first researcher [E.M.B.] coded the remaining transcripts.

The numbers and percentages of BCTs delivered in the consultation and the number and percentage of those BCTs recommended by the NCSCT treatment guidelines were examined using descriptive statistics, addressing Objective 1.

The data were hierarchically structured, with multiple patients per practitioner. Therefore, multilevel modelling (Level 1—patients (smokers); Level 2—practitioner) was used to simultaneously account for variability arising from the effect of any practitioner characteristics and known BCT content on smoking outcomes. Using SPSS v25, an intraclass correlation was used to estimate excess variability in individual outcomes by the community practitioner. Following this, the total number of BCTs delivered (Objective 2a), and BCTs as recommended by the treatment guidelines (Objective 2b), were added as level-one fixed effects in separate models, alongside potential level-1 correlates (including: gender, age, ethnicity, occupation, number of cigarettes smoked, time to first cigarette, cessation medications chosen, attendance at 4-week follow-up appointment, attendance at interval follow-up appointments) added as fixed-effect covariates. Changes in the deviance statistic and the effect size of the predictors were assessed. To calculate effect sizes, the beta estimate was raised to the exponent to obtain an odds ratio (*OR* = exp^*b*^^1^) [19]. Missing smoking status at follow-up was treated as smoking, in accordance with recommended abstinence measurement [20].

As part of an exploratory analysis, for each primary care practitioner, a delivery score was created for each BCT that they delivered at least once in an audio recorded consultation. The score was the average number of times each BCT was delivered across their audio-recorded consultations. For example, for a practitioner who delivered the BCT *goal setting* in three out of five audio-recordings, this would equate to a delivery score of 0.6 (3/5) for that BCT. This allowed an *a priori* exploratory analysis of 459 smokers with follow up data who had completed consultations with practitioners and had audio recorded at least three consultations but not with all included smokers (*n *= 149 audio recorded; *n *= 310 not audio recorded). The same multilevel modelling analytical procedure as described above for Objectives 2a and 2b was used to explore the relationship between practitioner delivery scores for each specific BCT and smoking abstinence for smoking outcomes (see Supplementary Material S2 for the association between specific BCTs and abstinence). Evidence indicates good within-practitioner BCT delivery consistency [11], and its use increases statistical power by increasing the sample size for this analysis. However, Objective (2c) was exploratory in using significance tests to generate rather than fully address hypotheses, and as such we accept an increased risk of Type 1 error as subsequent confirmation of any generated hypotheses would be required. Therefore, the results for Objective 2c should be interpreted with caution.

## Results

### Descriptives

#### Audio-recording

There were no statistically significant differences in abstinence between those participants (smokers) who were audio-recorded (*n *= 159) and trial participants (smokers) not audio-recorded (*n *= 443) at 4-weeks (25% [39/159] and 26% [113/443], respectively; *P* = .81), 8-weeks (45% [72/159] and 42% [185/443]; *P* = .44), or 6-months (9.4% [15/159] and 8.6% [38/443]; *P* = .74) (Chi-Square test).

#### Smoking abstinence

Out of 159 smokers whose initial consultation was audio-recorded, 149 (94%) had a complete and audible recording. Of these, 26% (38/149) were abstinent at 4-weeks, 46% (68/149) were abstinent at 8-weeks, and 10% (15/149) were abstinent at 6-months. Attrition rates were 73% (108/149) at 4-weeks, 83% (123/149) at 8-weeks, and 80% (119/149) at 6-months.

#### Duration of support

The average duration of audio-recorded smoking cessation support was ∼15 min (M= 14.9; SD = 7.4; range 3.0–39.0).

### Reliability of coding BCTs from audio-recorded transcripts

Using a random selection of transcripts (15/149; 10%), when all 121 potential BCTs were included in the denominator, coding reliability was very good (*k *= 0.82) with a percentage agreement of 94% (1710/1815). Coding reliability for only BCTs that had been coded at least once across the same 10% of transcripts was good (*k *= 0.78) with a percentage agreement of 90% (900/1005).

### BCT content of consultations

The mean number of BCTs delivered across all 149 consultations was 20.8 (SD = 6.7), which ranged from 8 to 51 BCTs per consultation. Eighty-five out of a possible 121 BCTs were delivered at least once across all 149 consultations with 17 delivered in at least 50% of the consultations (Table 1).

**Table 1:** Frequency of BCTs delivered in initial consultations in primary care.

BCTs delivered	% delivered; Included in treatment guidelines (Y/N) (NCSCT, 2012)
**11.1** Pharmacological support^a^	100%; Y
**RC10** Provide reassurance^b^	95%; N
**1.1** Goal setting (behavior)^a^	95%; Y
**RC1** Build general rapport^b^	91%; Y
**2.6** Biofeedback^a^	91%; N
**RC4** Explain expectations regarding the treatment program^b^	90%; Y
**RI1** Assess current and past smoking behavior^b^	87%; Y
**RI3** Assess past history of quit attempts^b^	82%; Y
**RI5** Assess nicotine dependence^b^	76%; Y
**5.1** Information about health consequences^a^	69%; N
**RC8** Elicit client views^b^	66%; Y
**RC3** Explain the purpose of CO monitoring^b^	63%; Y
**4.1** Instruction how to perform the behavior^a^	62%; N
**RC5** Offer/direct toward appropriate written materials^b^	61%; N
**3.1** Social support (unspecified)^a^	55%; Y
**RI7** Assess attitudes to smoking^b^	51%; N
**OTH1** Schedule follow-up appointment^c^	50%; N
**15.1** Verbal persuasion about capability^a^	46%; Y
**RI10** Assess physiological and mental functioning^b^	44%; N
**RC6** Provide information on withdrawal symptoms^b^	39%; Y
**8.2** Behavior substitution^a^	38%; N
**OTH2** Physiological effects of nicotine/smoking^c^	35%; N
**RD2** Emphasize choice^b^	34%; Y
**RC7** Use reflective listening^b^	32%; Y
**1.2** Problem solving^a^	32%; Y
**RI2** Assess current readiness and ability to quit^b^	31%; Y
**RD1** Tailor interactions appropriately^b^	30%; N
**RI6** Assess number of contacts who smoke^b^	28%; N
**RC9** Summarize information/confirm client decisions^b^	26%; Y
**12.4** Distraction^a^	24%; N
**BS13** Advise on methods of weight control^b^	22%; N
**12.1** Restructuring the physical environment^a^	22%; Y
**1.4** Action planning^a^	21%; Y
**BM13** Create or reinforce negative associations^b^	20%; N
**13.2** Framing/reframing^a^	19%; N
**RI8** Assess level of social support^b^	15%; N
**RI9** Explain how tobacco dependence develops^b^	15%; Y
**4.2** Information about antecedents^a^	15%; N
**7.1** Prompts/cues^a^	14%; N
**5.3** Information about social and environmental consequences^a^	13%; N
**12.3** Avoidance/reducing exposure to cues for the behavior^a^	13%; Y
**12.5** Adding objects to the environment^a^	13%; N
**5.2** Salience of consequences^a^	10%; N
**A5** Give options for additional and later support^b^	9%; Y
**1.3** Goal setting (outcome)^a^	9%; N
**2.4** Self-monitoring of outcomes of behavior^a^	9%; N
**6.1** Demonstration of the behavior^a^	9%; Y
**4.3** Re-attribution^a^	8%; N
**4.5** Tell to act^a^	8%; N
**5.6** Information about emotional consequences^a^	8%; N
**10.7** Self-incentive^a^	8%; N
**12.2** Restructuring the social environment^a^	8%; N
**13.5** Identity associated with changed behavior^a^	6%; Y
**15.4** Self-talk^a^	6%; N
**2.2** Feedback on behavior^a^	5%; N
**15.3** Focus on past success^a^	5%; Y
**RD3** Promote engagement with the program^b^	4%; N
**BM10** Explain the importance of abrupt cessation^b^	3%; Y
**RI4** Assess withdrawal symptoms^b^	3%; N
**8.6** Graded tasks^a^	3%; N
**5.4** Monitoring of emotional consequences^a^	2%; N
**8.4** Habit reversal^a^	2%; N
**9.1** Credible source^a^	2%; N
**9.2** Pros and cons^a^	2%; Y
**11.2** Reduce negative emotions^a^	2%; N
**A4** Ask about experiences of stop smoking medication that the smoker is using^b^	1%; N
**1.7** Review outcome goal^a^	1%; N
**1.9** Commitment^a^	1%; Y
**2.3** Self-monitoring of behavior^a^	1%; Y
**2.7** Feedback on outcome(s) of behavior^a^	1%; N
**3.2** Social support (practical)^a^	1%; N
**3.3** Social support (emotional)^a^	1%; N
**4.4** Behavioral experiments^a^	1%; N
**5.5** Anticipated regret^a^	1%; N
**8.1** Behavioral practice/rehearsal^a^	1%; N
**8.3** Habit formation^a^	1%; N
**9.3** Comparative imagining of future outcomes^a^	1%; N
**10.1** Material incentive (behavior)^a^	1%; N
**10.5** Social incentive^a^	1%; N
**10.8** Incentive (outcome)^a^	1%; N
**10.9** Self-reward^a^	1%; N
**13.1** Identification of self as a role model^a^	1%; N
**13.3** Incompatible beliefs^a^	1%; N
**15.2** Mental rehearsal of successful performance^a^	1%; N
**16.2** Imaginary reward^a^	1%; N
**1.5** Review behavior goals^a^	0%; N
**1.6** Discrepancy between current behavior and goal^a^	0%; N
**1.8** Behavioral contract^a^	0%; N
**2.1** Monitoring of behavior by others with feedback^a^	0%; N
**2.5** Monitoring of outcome(s) of behavior by others without feedback^a^	0%; N
**6.2** Social comparison^a^	0%; N
**6.3** Information about others’ approval^a^	0%; N
**7.2** Cue signaling reward^a^	0%; N
**7.3** Reduce prompts/cues^a^	0%; N
**7.4** Remove access to the reward^a^	0%; N
**7.5** Remove aversive stimulus^a^	0%; N
**7.6** Satiation^a^	0%; N
**7.7** Exposure^a^	0%; N
**7.8** Associate learning^a^	0%; N
**8.5** Generalization of target behavior^a^	0%; N
**10.2** Material reward (behavior)^a^	0%; Y
**10.3** Non-specific reward^a^	0%; N
**10.4** Social reward (behavior)^a^	0%; N
**10.6** Non-specific incentive^a^	0%; N
**10.10** Reward (outcome)^a^	0%; N
**10.11** Future punishment^a^	0%; N
**11.3** Conserving mental resources^a^	0%; N
**11.4** Paradoxical instructions^a^	0%; N
**13.4** Valued self-identity^a^	0%; N
**14.1** Behavior cost^a^	0%; N
**14.2** Punishment^a^	0%; N
**14.3** Remove reward^a^	0%; N
**14.4** Reward approximation^a^	0%; N
**14.5** Rewarding completion^a^	0%; N
**14.6** Situation-specific reward^a^	0%; N
**14.7** Reward incompatible behavior^a^	0%; N
**14.8** Reward alternative behavior^a^	0%; N
**14.9** Reduce reward frequency^a^	0%; N
**14.10** Remove punishment^a^	0%; N
**16.1** Imaginary punishment^a^	0%; N
**16.3** Vicarious consequences^a^	0%; N

a BCTs from the IC SMOKE/BCTTv1 (de Bruin *et al*., unpublished work).^15^

b BCTs from the smoking-specific taxonomy.^14^

c BCTs from PPI inclusions.

The six most commonly delivered BCTs, delivered in at least 90% of consultations were: *Pharmacological support* (100%, *n *= 149), *Provide reassurance* (95%, *n *= 142), *Goal setting (behavior)* (95%, *n *= 142), *Build general rapport* (91%, *n *= 136), *Biofeedback* (91%, *n *= 136), and *Explain expectations regarding the treatment program* (90%, *n *= 134) (Table 1).

In terms of BCT delivery recommended by the NCSCT treatment guidelines, 29 out of 30 (96.7%) were delivered in at least one of the 149 consultations (M = 13.4; SD = 2.76), which ranged from 6 to 22 recommended BCTs per consultation. Ten out of the recommended 30 (33%) BCTs were delivered in at least 50% of the consultations. Of these recommended BCTs, *Material reward* was not delivered in any consultation, with *Self-monitoring of behavior, Commitment*, and *Pros and cons* only delivered in 1%–2% of consultations (see Table 1 for the percentage delivery of all recommended BCTs).

A *post hoc* Pearson correlation analysis found that the total number of BCTs delivered was associated with the duration of the consultation (*r*[147] = 0.63, *p *< .001).

### Association between number of BCTs delivered and smoking abstinence

The total number of BCTs delivered in the initial consultation was not significantly associated with the primary smoking abstinence outcome at 8-week (*OR *= 1.00, 95% *CI* = 0.98–1.01; *p *= .95), or the secondary abstinence outcomes at 4-week (*OR *= 1.01, 95% *CI* = 1.00–1.03; *p *= .07), and 6-month (*OR *= 1.00, 95% *CI* = 0.99–1.01; *p *= .82) follow-up (Table 2).

**Table 2:** Multilevel modelling results for the association between the total number of BCTs delivered and smoking abstinence at 8-week, 4-week, and 6-month follow-up.

	Self-reported abstinence at 8-weeks (primary outcome)		CO verified abstinence at 4-weeks			Self-reported abstinence at 6-months		
Variable	Estimate	Odds Ratio (95% *CI*)	Estimate	Odds Ratio (95% *CI*)		Estimate		Odds Ratio (95% *CI*)
Trial condition^a^	−0.03	0.97 (0.81–1.17)	0.04		1.04 (0.88–1.23)	−0.15	0.86 (0.76–0.98)	
Sex^a^	−0.04	0.96 (0.79–1.18)	−0.12		0.89 (0.74–1.07)	−0.005	0.99 (0.86–1.14)	
*Total BCTs*	−0.0005	1.00 (0.98–1.01)	0.01		1.01 (1.00–1.03)	−0.001	1.00 (0.99–1.01)	
**Potential level one confounders**								
Age	0.01	1.01 (1.00–1.02)	0.002		1.00 (0.99–1.01)	0.003	1.00 (1.00–1.01)	
Daily number of cigarettes	−0.001	1.00 (0.99–1.01)	−0.003		1.00 (0.99–1.01)	−0.002	1.00 (0.99–1.01)	
Time to first cigarette	−0.04	0.96 (0.85–1.08)	0.01		1.01 (0.91–1.13)	−0.03	0.97 (0.89–1.05)	
Ethnicity	−0.003	1.00 (0.94–1.06)	−0.04		0.96 (0.91–1.02)	0.01	0.99 (0.95–1.04)	
Occupation	0.02	1.02 (0.97–1.06)	0.06		1.06 (1.02–1.10)	0.02	1.02 (0.99–1.05)	
Medication^a^	−0.41	0.66 (0.50–0.88)	0.009		1.01 (0.77–1.32)	−0.08	0.92 (0.74–1.15)	
4-week follow-up attendance	0.26	1.30 (1.02–1.65)	0.29		1.33 (1.07–1.67)	0.06	1.06 (0.89–1.26)	
Number of clinic visits at 4-week follow-up	−0.01	0.99 (0.92–1.07)	0.02		1.02 (0.95–1.10)	0.01	1.01 (0.96–1.07)	

a The reference category for binary variables is the category with a higher numeric value. The above variables were coded as follows: Condition (control = 0, Intervention = 1), Sex (Male = 1, Female = 2), Time of first cigarette (1 = Within 30 min, 2 = 31–59 min, 3 = 1–2 h, 4 = >2 h), Ethnicity (1 = British, 2 = Irish, 3 = Other White British, 4 = Caribbean, 5 = African, 6 = Other Black background, 7 = Indian, 8 = Pakistani, 9 = Bangladeshi, 10 = Other Asian background, 11 = Mixed White and Black Caribbean, 12 = Mixed White and Black African, 13 = Mixed White and Black Asian, 14 = Mixed other, 15 = Chinese, 16 = Other ethnic group), Occupation (1 = Full time student, 2 = Never worked/long term unemployed, 3 = Retired, 4 = Sick/disabled/unable to work, 5 = Home carer, 6 = Managerial/professional, 7 = Intermediate, 8 = Routine and Manual), Medication (0 = None, 1 = Yes).

The total number of recommended BCTs delivered in the initial consultation was not significantly associated with the primary abstinence outcome at 8-weeks, (*OR *= 0.99, 95% *CI* = 0.96–1.02; *p *= .39), or abstinence at 6-months (*OR *= 1.00, 95% *CI* = 0.98–1.02; *p *= .88). However, it was positively associated with abstinence at 4-weeks (*OR *= 1.03, 95% *CI* = 1.00–1.06; *p *= .03) (Table 3).

**Table 3:** Multilevel modelling results for the association between the recommended BCTs delivered and smoking abstinence at 8-week, 4-week, and 6-month follow-up.

	Self-reported abstinence at 8-weeks (primary outcome)		CO verified abstinence at 4-weeks		Self-reported abstinence at 6-months	
Parameter	Estimate	Odds ratio (95% *CI*)	Estimate	Odds ratio (95% *CI*)	Estimate	Odds ratio (95% *CI*)
Trial condition^a^	−0.04	0.96 (0.80–1.15)	0.04	1.04 (0.88–1.23)	−0.15	0.86 (0.76–0.98)
*BCTs included in NCSCT treatment guidelines*	−0.01	0.99 (0.96–1.02)	0.03	1.03 (1.00^b^–1.06)	−0.002	1.00 (0.98–1.02)
**Potential level one confounders**						
Sex	−0.02	0.98 (0.81–1.20)	−0.12	0.89 (0.74–1.07)	−0.01	0.99 (0.86–1.14)
Age	0.01	1.01 (1.00–1.02)	0.001	1.00 (0.99–1.01)	0.003	1.00 (1.00–1.01)
Daily number of cigarettes	−0.001	1.00 (0.99–1.01)	−0.003	1.00 (0.99–1.01)	−0.002	1.00 (0.99–1.01)
Time to first cigarette	−0.03	0.97 (0.86–1.09)	0.004	1.00 (0.90–1.12)	−0.03	0.97 (0.89–1.05)
Ethnicity	0.0005	1.00 (0.94–1.07)	−0.04	0.96 (0.91–1.02)	−0.01	0.99 (0.95–1.04)
Occupation	0.01	1.01 (0.97–1.06)	0.06	1.06 (1.02–1.1)	0.02	1.02 (0.99–1.05)
Medication	−0.41	0.66 (0.5–0.88)	0.02	1.02 (0.78–1.33)	−0.08	0.92 (0.74–1.14)
4-week follow-up attendance	0.26	1.29 (1.02–1.64)	0.28	1.33 (1.07–1.66)	0.06	1.06 (0.89–1.26)
Number of clinic visits at 4-week follow-up	−0.01	0.99 (0.92–1.07)	0.03	1.03 (0.96–1.10)	0.01	1.01 (0.96–1.07)

a The reference category for binary variables is the category with a higher numeric value. The above variables were coded as follows: Condition (control = 0, Intervention = 1), Sex (Male = 1, Female = 2), Time of first cigarette (1 = Within 30 min, 2 = 31–59 min, 3 = 1–2 h, 4 = >2 h), Ethnicity (1 = British, 2 = Irish, 3 = Other white British, 4 = Caribbean, 5 = African, 6 = Other black background, 7 = Indian, 8 = Pakistani, 9 = Bangladeshi, 10 = Other Asian background, 11 = Mixed White and Black Caribbean, 12 = Mixed White and Black African, 13 = Mixed White and Black Asian, 14 = Mixed other, 15 = Chinese, 16 = Other ethnic group), Occupation (1 = Full time student, 2 = Never worked/long term unemployed, 3 = Retired, 4 = Sick/disabled/unable to work, 5 = Home carer, 6 = Managerial/professional, 7 = Intermediate, 8 = Routine and Manual), Medication (0 = None, 1 = Yes).

b The lower confidence interval is above 1 but when rounded up to two decimal places it indicates this value is 1 exactly.

### Exploratory analysis: Associations between specific BCTs and smoking abstinence

Exploratory analyses identified one significant positive association between practitioner BCT delivery scores and abstinence at 8-weeks for *Use reflective listening* (*OR *= 1.57, 95% *CI* = 1.00–2.48; *p *= .050). Another BCT delivery score and abstinence showed a borderline statistically significant negative association, *Self-monitoring of behavio*r (*OR *= 0.35, 95% *CI* = 0.12–1.02; *p *= .053). No additional practitioner BCT delivery scores were found to be associated with smoking abstinence. In many cases, models would not converge due to low event or delivery rates.

## Discussion

This is the first study to use individual-level data from UK primary care smoking cessation consultations to identify the BCTs delivered and investigate whether their delivery is associated with abstinence. Key findings were that a large number (85) of different BCTs were delivered across all consultations, and that the total number of BCTs delivered was not associated with abstinence, but the number of recommended BCTs was associated with short term abstinence. In addition, a minimum of 6 of the 30 BCTs recommended in guidance were delivered in all consultations, with 10 or more delivered in at least half of all consultations.

The findings support prior expectations that the recommended cessation BCTs delivered by primary care practitioners varies considerably and is lower than when delivered by specialist stop smoking practitioners, those whose only role is specific to delivering smoking cessation support. In our study, the average pre-quit consultation included 43% (13/30) of recommended BCTs, whereas this was found to be 58% for specialist stop smoking programs in England, according to one study [11]. A lack of consistency in BCT delivery with regards to treatment guidelines has also been identified within non-specialist cessation services, such as primary care, in other countries such as Belgium [21] and Greece [22]. Reasons for this identified inconsistency to guidelines included a lack of time [21] and differences in client characteristics [22].

Consistent with previous research [13], we did not identify a relationship between the total number of BCTs delivered by practitioners in the consultation and smoking abstinence. This implies that the quantity of BCTs is not important for achieving abstinence. In contrast, we identified an association between the total number of recommended BCTs in national guidance delivered in the consultation and short-term, but not long-term, abstinence. Given the small effect size, this should not be interpreted as a dose–response relationship (i.e., more BCTs increase abstinence). It is plausible that the association may be driven by a smaller subset of effective BCTs, not identified in this study. In addition, when considering factors of lack of time [21] and differences in client characteristics [22] identified in primary care, the mean (13) and range of guideline recommended BCTs (6–22) delivered in the current study may be more realistic than the 30 recommended in treatment guidelines.

Two BCTs, based on a practitioner’s BCT delivery score, showed association/borderline association with abstinence; *Use reflective listening* (positive association) and *Self-monitoring of behavior* (borderline negative association). Caution should be applied to these findings given their exploratory nature and aim to generate hypotheses for future work. While these associations could represent evidence of benefit or harm for these specific BCTs, an alternative explanation for such associations is that BCTs may be delivered based on the practitioners’ perceived need of their clients. This is also a potential explanation for the lack of association between the number of BCTs delivered and abstinence—practitioners may deliver more BCTs to those that they anticipate will require greater support due to characteristics (e.g., tobacco dependence) or circumstances (e.g., lack of social support) that will otherwise reduce their chances of quitting successfully. Furthermore, while the potential practitioner-level influence was accounted for in the multilevel analysis, findings may still reflect features of practitioners that are associated with BCT delivery.

## Strengths and limitations

Previous research that has identified BCTs in smoking cessation, in research interventions and treatment guidelines [13, 12], have used a previous shorter version of the BCT taxonomy [14]. Therefore, use of a coding tool that includes 121 BCTs (i.e., smoking-specific BCTs, smoking cessation modified general BCTs plus BCTs derived through public and patient involvement), excluding those that overlapped, within the present study adds to the existing evidence base. This allows us to understand that a wide range of BCTs are used in initial smoking cessation consultations within primary care, though we are less clear on whether training was delivered, or whether training could mean that the BCTs become more effective toward both short- and long-term abstinence.

Attempts to identify effective components of routine care come with a number of challenges and are important considerations of future research. First, we chose *a priori* to use the self-reported 8-week abstinence outcome as the primary smoking outcome because the biochemically verified abstinence outcome at 4-weeks was undertaken routinely by practitioners, rather than the research team, and consequently had a sub-optimal response rate. While the implications of not having a biochemically validated outcome measure may be small given previous evidence that self-reported measures are highly accurate for smokers who are not adolescents or considered to be high-risk smokers [23], this still remains a limitation. Future work in this area should strive, where feasible, to biochemically validate long-term smoking outcomes.

Second, as the individual-level audio-recordings of consultations included in the present study were collected from cessation advice delivered in primary care, the findings may not be generalizable to other practitioners (e.g., specialist) or behavioral support (e.g., alcohol treatment). Relatedly, the data were also collected in the context of a trial [17], so the findings may not be generalizable to a non-trial routine practice context and practitioners’ routine care may have been influenced by the research activities, which may have contributed to some practitioners not audio-recording consultations, as requested. In addition, there is the possibility that specific BCTs would be differentially effective or influential at different stages of behavior change, such as initiating versus maintaining [24]. While primary care practitioners are expected to deliver the majority of cessation support during the initial consultation [7, 11], investigating the effect of BCTs delivered at follow-up appointments on abstinence would help guide treatment guidelines further, but was not assessed as part of this study.

Third, the analyses were conducted at the level of practitioner behavior, examining associations between general patterns of practitioner delivery and patient outcomes. As such, these findings do not support inferences regarding the effects of specific, or combinations of BCTs, on abstinence, particularly given the intercorrelated nature of BCT delivery, which can limit the ability to isolate independent effects.

In addition, understanding why practitioners delivered their chosen BCTs including the total number is unclear. This remains a challenge of using observational data to assess the effectiveness of content where practitioners can tailor support according to their perceived clients’ needs, as well as the time available within that consultation. It could be valuable for future research to consider controlled studies in which practitioners are trained and deliver different subsets of BCTs, dependent on such factors. Furthermore, there is a lack of variance for a few BCTs (e.g., *Pharmacological support*), which precludes assessing their specific influence on outcomes.

Fourth, the quality of the delivery of specific BCTs or those considered to be of prime importance [11, 12] were not assessed in the current study. Quality could include the duration of specific BCT delivery, which would likely address the challenge of incorporating duration of overall support delivery into predictive models while avoiding measurement overlap with the number of BCTs delivered. This is therefore a limitation of the present study as we may have missed an active dimension of support effectiveness. Given the complexity involved in creating a reliable quality assessment for each or key BCTs [25], this is work for a future project. We may also have missed out on other important features of support delivery and receipt, including patient communication and duration of delivery, which was not included in models due to anticipated measurement overlap with the number of BCTs. However, the use of multilevel models should have at least accounted for the influence of consistent practitioner characteristics.

Finally, the delivery score was based on the expectation that practitioners delivered similar BCTs to different patients, but we were not able to determine this, and so the findings from the delivery score analysis should be treated with caution.

## Implications for future research

The findings indicate that at least six (out of 30) BCTs recommended in treatment guidelines are routinely delivered, with 10 BCTs delivered in at least half of initial consultations. There are likely multiple reasons why more recommended BCTs are not delivered, including low self-efficacy, lack of training, or practical factors such as a lack of time or resources, commonly experienced in primary care [21, 25]. Future research exploring why specific BCTs, including those that are recommended in treatment guidelines, are not delivered in the initial consultation within primary care could help to inform training and potentially the refinement of guidelines to ensure that recommended BCTs are fit for purpose. If further training and feedback are required, this may also address any insufficiencies in training that could otherwise impact on practitioner’s self-efficacy to deliver appropriate cessation support [26]. If future research identifies that practitioners do not feel certain BCTs are appropriate or viable to deliver during cessation consultations, it may highlight the need for support adjuncts, such as digital interventions [17], that could supplement the content provided within consultations in primary care.

The limited evidence for a benefit of BCT delivery, including those recommended in treatment guidelines, and cessation beyond a short-term effect could indicate that other non-BCT-related factors are key for supporting abstinence in the long-term. Future research may therefore benefit from the use of more advanced analysis approaches, such as machine learning, to explore whether specific combinations of subsets of BCTs differentially contribute to abstinence outcomes. Notably, the study identified that a number of key recommended BCTs considered to be effective [7, 12], including *Assessing current readiness and ability to quit*, *Problem solving*, *Action planning*, and *Restructuring the physical environment*, were rarely delivered within the initial consultation. Although it is possible that some key BCTs could be more impactful if delivered during follow-up appointments, given the relatively low attendance at follow-up consultations in primary care [17], optimizing the initial consultation to address the identified treatment gaps could help improve cessation outcomes.

## Conclusion

The present study is the first to use individual-level recordings to assess BCT delivery in primary care and relate these to short- and long-term abstinence outcomes. The study findings suggest that the total number of BCTs may not be important for long-term abstinence, though total number of BCTs recommended by treatment guidelines may help improve short-term abstinence. The study also identified that one-third of BCTs recommended by treatment guidelines are delivered routinely (in at least half of initial consultations) in primary care, despite factors such as time constraints in this setting. The findings can help inform treatment guideline updates for the delivery of cessation advice as well as contributing to improving training for primary care practitioners.

## Supplementary Material

ibag035_Supplementary_Data

## Transparency statement

- Study registration: This study was not formally registered.
- Analytic plan registration: The analytic plan was not formally registered.
- Availability of data: De-identified data from this study is not available in public archive. De-identifiable data from this study will be made available on reasonable request (as allowable according to ethical standards) by emailing the corresponding author.
- Availability of analytic code: Analytic code used to conduct the analyses presented in this study are not available in public archive. This may be available by emailing the corresponding author.
- Availability of materials: Materials used to conduct the study are available on reasonable request.
