# Supplementary material for: Behavior change technique delivery during routine smoking cessation advice in primary care and associations with abstinence
Source: Transl Behav Med. 2026 Jul 3;16(1):ibag035. doi: 10.1093/tbm/ibag035 (PMC13331279; doi:10.1093/tbm/ibag035)
Supplement: ibag035_Supplementary_Data [file ibag035_supplementary_data.docx]

**Supplementary material S1.**

**Coding framework for identifying behavior change techniques and competences from transcripts of stop smoking interactions**

Coding tool used to code BCTs to audio-recorded initial smoking cessation consultations

This coding framework combines two previous behavior change technique taxonomies (BCTTs). The first one (Michie, Churchill, & West, 2011) provided for use in coding smoking specific situations (pages 3-7) and the second one (IC SMOKE/BCTTv1, de Bruin et al. Unpublished; Michie et al., 2013) provided for use in coding a range of behaviors (pages 8-33) have been included to ensure the most comprehensive list of BCTs and competences available.

Both taxonomies have been adapted for their use in coding BCTs/competences from transcripts of stop smoking interactions in Primary Care.

**The first taxonomy (pages 3-7) consists of four columns as follows:**

| Column 1: | Provides the code to be used in the coding template when assigning each BCT/competency (e.g., BM_10). This is followed by the definition for this BCT/competency |
| --- | --- |
| Column 2: | Provides a smoking specific example, which includes the specificity of this BCT/competency to help with identifying and coding the correct BCT/competency |
| Column 3: | Provides the codes of any foreseeable overlap between this taxonomy and the IC SMOKE taxonomy (pages 8-33). If a BCT/competency is coded that has been associated with possible overlap, please include this overlap in brackets when coding the transcripts, in to the coding template (e.g., (6.3)). This will help when coding BCTs/competencies using the second taxonomy |

**The second taxonomy (pages 8-33) consists of three columns as follows:**

| Column 1: | Provides the code to be used in the coding template when assigning each BCT (e.g., 1.8). This is followed by the definition for this BCT |
| --- | --- |
| Column 2: | Provides a smoking specific example to help with identifying and coding the correct BCT |
| Column 3: | Provides the codes of any foreseeable overlap between this taxonomy and the smoking specific taxonomy (pages 3-7). BCTs that have been identified to overlap with those found in the smoking specific taxonomy have been identified using red font (to help with ease of coding across both taxonomies) |

Note: Please code all BCTs/competences present for just the practitioner only. However, if there are instances by which you feel it would be beneficial to also code the BCTs present within the statements made by the client, please flag these up with the team before coding these.

**Instructions for use of the coding framework**

1. **Read the entire transcript**

Reading the transcript first, in its entirety, will help you to get an overview of the length and depth of each session delivered, but also how long it may take to code each transcript. It may also help you to highlight some specific/repeated BCTs/competences from the discussions.

1. **Read the entire transcript in detail and assign a code where applicable**

The coding template provides the whole transcript, broken down into segments of speech between: (1) the practitioner, and (2) the client. Before assigning the code per BCT/competency within each segment of speech, re-read the definition of the code. If the code chosen fits the definition, write the BCT/competency code into the code column of the coding template (use the code only, the name of the BCT/competency, or definition are not required). If a code is identified as overlapping with another BCT/competency please present both codes, in separate cells. This will help to establish that the code may have already been identified.

1. **Additional comments**

If a section of the speech appears relevant to smoking/smoking cessation but is unclear which BCT/competency code to assign, please make notes about this in the comments section. Also note any other issues (e.g., sections of the transcript that appear to be missing).

1. **Some codes may be different from those present in the previous versions of the taxonomies used**

Please code the BCTs/competences as they are found within the coding framework below. Some codes will have underscores in (e.g., BM_10 or 10_10) that were not associated with the previous versions of the taxonomies that have been adapted for this project. This has been used to develop a formula to establish overall calculations once the coding is complete (e.g., for reliability measures).

**Additional guidance**

1. **Only code what is present in the conversation**

Only code the BCT/competency if this is present in the conversation that you are coding. Do not code a BCT/competency that you assume should be present or that you think would follow on from that conversation. If any speech is interrupted during the transcription that forms only part of a BCT/competency, do not code. If in doubt, make a note of this uncertainty in the comments section.

1. **Multiple BCTs/competencies present in the same statement**

There may be instances in which the delivery of two or more BCTs/competencies are present within one segment of text. For example, “A craving can last up to 10 minutes, so instead you should clean your teeth then come downstairs and make a cup of tea”. The practitioner is providing 4.2 and 12.3. Regardless of whether these BCTs/competences are delivered together or separately within a section, please code all identified BCTs/competences in separate cells.

1. **Avoid bias**

Code each and every instance of a BCT/competency, even if a specific BCT has been identified and coded previously in the same transcript. You should also aim to avoid making inferences based on your perceptions of skill/quality of the delivery of the BCTs/competencies/sessions.

| **Page** | **Grouping and BCTs** |
| --- | --- |
| 5 | **A. Specific focus on behavior (B) addressing motivation (M)** |
|  | **BM_10** Explain the importance of abrupt cessation |
|  | **BM_13** Create or reinforce negative associations |
|  |  |
| 5 | **B. Specific focus on behavior (B) maximising self-regulation capacity/skills (S)** |
|  | **BS_13** Advise on methods of weight control |
|  |  |
| 5 | **C. Promote adjuvant activities (A)** |
|  | **A4** Ask about experiences of stop smoking medication that the smoker is using |
|  | **A5** Give options for additional and later support |
|  |  |
| 5 | **D. General aspects of the interaction (R.) focusing on: delivery of the intervention (D), information gathering (I), or general communication (C.)** |
|  | **RD1** Tailor interactions appropriately |
|  | **RD2** Emphasise choice |
|  | **RD3** Promote engagement with the programme |
|  | **RI1** Assess current and past smoking behavior |
|  | **RI2** Assess current readiness and ability to quit |
|  | **RI3** Assess past history of quit attempts |
|  | **RI4** Assess withdrawal symptoms |
|  | **RI5** Assess nicotine dependence |
|  | **RI6** Assess number of contacts who smoke |
|  | **RI7** Assess attitudes to smoking |
|  | **RI8** Assess level of social support |
|  | **RI9** Explain how tobacco dependence develops |
|  | **RI_10** Assess physiological and mental functioning |
|  | **RC1** Build general rapport |
|  | **RC3** Explain the purpose of CO monitoring |
|  | **RC4** Explain expectations regarding the treatment programme |
|  | **RC5** Offer/direct towards appropriate written materials |
|  | **RC6** Provide information on withdrawal symptoms |
|  | **RC7** Use reflective listening |
|  | **RC8** Elicit client views |
|  | **RC9** Summarise information/confirm client decisions |
|  | **RC_10** Provide reassurance |
|  | **OTH1** Schedule follow-up appointment |
|  | **OTH2** Physiological effects of nicotine/smoking |

| **Smoking specific TAXONOMY – Techniques and competences** | **Example** | **Overlap with BCTs present in BCTTv1** | **Further associations** |
| --- | --- | --- | --- |
| **BM_10** Explain the importance of abrupt cessation | Encourage the client to stop abruptly and explain why it is better to do so rather than cut down gradually. | ***4.2 Information about antecedents*** | In NCSCT guidance |
| **BM_13** Create or reinforce negative associations | Present descriptions or labels to the client that aim to generate negative emotional associations with smoking other than by providing information about the negative consequences of smoking. |  |  |
| **BS_13** Advise on methods of weight control | Suggest that the client eats healthy snacks when they get tempted to smoke instead of eating sweets.  *Note: Discussing changes in weight/metabolism as a result of smoking cessation should be coded as 5.1.* |  |  |
| **A4** Ask about experiences of stop smoking medication that the smoker is using | Ask the client, which stop smoking medications they are using and how they are finding them (e.g., sustained quit attempt, allergic reaction, taste). |  | Associated with abstinence (West et al., 2010) |
| **A5** Give options for additional and later support | Provide information to the client about options for additional support outside of the stop smoking programme and where these are available. | ***3.1 Social support (unspecified)*** | In NCSCT guidance  Associated with abstinence (West et al., 2010) |
| **RD1** Tailor interactions appropriately | Ask the client about previous relapses to smoking and help the client to understand why this happened.  *Note: Make sure there is sufficient discussion between the practitioner and the client before coding this BCT.* |  | In NCSCT guidance |
| **RD2** Emphasise choice | Advise the client that they have to choose which stop smoking medication to use, but that you can help them to make an informed choice.  Advise the client to choose a quit date that works best around their upcoming commitments.  *Note: There needs to be sufficient evidence that the practitioner is clearly offering a choice between options or highlighting the aspects that the client may want to take into account in order to make a decision.* |  | In NCSCT guidance |
| **RD3** Promote engagement with the programme | Provide information or encouragement to the client aimed at promoting the use of the programme now or in the future.  *Note: There needs to be sufficient evidence of active effort on part of the practitioner to promote engagement rather than just indicating a follow-up appointment.* |  |  |
| **RI1** Assess current and past smoking behavior | Assess the amount of cigarettes smoked, age smoking was initiated, patterns of smoking behaviors.  *Note: This should also be coded for instances of “How many cigarettes do you smoke a day?”, “How soon after waking do you smoke your first cigarette of the day?”.* |  | In NCSCT guidance |
| **RI2** Assess current readiness and ability to quit | Ask the client if they are ready to stop smoking for good.  Ask the client if this is the right time to try to stop smoking.  *Note: Only code RI2 when there is sufficient evidence of the practitioner assessing the clients readiness/ability (e.g., How determined are you to do this?).* |  | In NHS 2003 standard treatment manual  In NCSCT guidance |
| **RI3** Assess past history of quit attempts | Ask the client if they have ever quit smoking before and how long for.  *Note: This should include any discussion of past experiences of quitting including medication or environmental restructuring.* |  | In NCSCT guidance |
| **RI4** Assess withdrawal symptoms | Ask the client if they regularly have urges to smoke or have noticed any differences in their mood. |  |  |
| **RI5** Assess nicotine dependence | Ask the client how soon after they wake up, they smoke a cigarette.  Ask the client how many cigarettes they smoke on average each day.  *Note: This should also be coded for instances of “How many cigarettes do you smoke a day?”, “How often do you get urges to smoke?”, “How soon after waking do you smoke your first cigarette of the day?”.* |  | In NCSCT guidance |
| **RI6** Assess number of contacts who smoke | Ask the client if they live with anyone else who smokes.  Ask the client if anyone within his or her wider social circle smokes. |  |  |
| **RI7** Assess attitudes to smoking | Ask the client why they want to quit smoking this time.  Ask the client to specify the costs and benefits of smoking. | ***4.3 Re-attribution*** | In NCSCT guidance |
| **RI8** Assess level of social support | Ask the client if they have family and/or friends who will be supportive of them during this quit attempt. |  |  |
| **RI9** Explain how tobacco dependence develops | Inform the client that repeated exposure to nicotine through smoking causes the brain to release dopamine, which can make smoking feel pleasurable.  *Note: Code this only if the practitioner discusses nicotine dependence without the descriptions of medication (see OTH2).* |  | In NCSCT guidance |
| **RI_10** Assess physiological and mental functioning | Ask the client about any current or previous health diagnoses that may impact the advice offered regarding the most appropriate stop smoking medications. |  |  |
| **RC1** Build general rapport | Establish a positive, friendly, and professional relationship with the client. This must be in regard to the behavior (smoking/smoking cessation).  *Note: This includes saying thank you or providing gratitude.* |  | In NCSCT guidance |
| **RC3** Explain the purpose of CO monitoring | Explain that cigarettes contain carbon monoxide, a poisonous gas that deprives the body of oxygen and that a simple breath test can be carried out to determine the client’s current CO levels. |  | In NHS 2003 standard treatment manual  In NCSCT guidance  Associated with abstinence (West et al., 2010) |
| **RC4** Explain expectations regarding the treatment programme | Inform the client that they are expected to have quit smoking within 14 days of the first stop smoking session.  *Note: Also, code RC4 if the practitioner explains expectations outside of the treatment programme (e.g., also requires motivation from the client).* |  | In NHS 2003 standard treatment manual  In NCSCT guidance |
| **RC5** Offer/direct towards appropriate written materials (not including the use of materials as props within the consultation) | Provide the client with a booklet with further information on avoiding smoking triggers.  Inform the client that they can pick-up a booklet to help with stopping smoking at the reception of their GP surgery.  *Note: This should be coded only if there is evidence that the practitioner has provided the materials to the client which focuses on avoiding smoking triggers. Do not code if materials are used as a prop or guide.* |  |  |
| **RC6** Provide information on withdrawal symptoms | Provide information to the client on the symptoms of nicotine withdrawal, including commonality, duration, and how to alleviate them. |  | In NHS 2003 standard treatment manual  In NCSCT guidance  Associated with abstinence (West et al., 2010) |
| **RC7** Use reflective listening | Provide agreement (e.g., verbal, non-verbal) on the client’s views of stop smoking medications, summarising the discussion afterwards to confer understanding. |  | In NCSCT guidance  Associated with abstinence (West et al., 2010) |
| **RC8** Elicit client views | Ask the client their views on the stop smoking support received.  Prompt the client to express how they feel about the decisions that have been made (e.g., quit date, pharmcotherapy).  *Note: Only code if there is clear evidence of the practitioner prompting the views of the client regarding making decisions or supporting options.* |  | In NCSCT guidance  Associated with abstinence (West et al., 2010) |
| **RC9** Summarise information/confirm client decisions | Summarise what you expect the client to do before the next appointment (e.g., collect medication, quit).  Confirm which medications the client has chosen.  *Note: Code this only when there is sufficient evidence that discussions have been summarised (e.g., in a list like form), or repetition of decisions is present in a questionable way (e.g., You said you want to try Champix, and you have used this before?)* |  | In NCSCT guidance  Associated with abstinence (West et al., 2010) |
| **RC_10** Provide reassurance | Tell the client that it is common to be nervous about quitting smoking.  Inform the client that their carbon monoxide level may be high this time, but that this will come down to a non-smokers level, just hours after their last cigarette. | ***3.1 – Social support (unspecified)***  ***15.1 – Verbal persuasion about capability*** | In NCSCT guidance  Associated with abstinence (West et al., 2010) |
| **OTH1** Schedule follow-up appointment | Arrange or invite the client to arrange a follow-up appointment to assess changes in their smoking behavior (e.g., if they have quit).  *Note: Code also if the practitioner is inviting the client to make a follow-up appointment – even if a specific date is not recorded.* |  |  |
| **OTH2** Physiological effects of smoking cessation medications | Inform the client of the saw-tooth pattern of constant change to the levels of nicotine in the brain that drives the addiction, to help in explaining how the different stop smoking medications work (e.g., nicotine replacement therapy).  *Note: This should be coded instead of RI9 in instances where the practitioner is discussing/explaining the physiological effects of smoking cessation medications (e.g., how these work within the body to reduce cravings).* |  |  |

| **BCT** | **Example** | **Overlap** |
| --- | --- | --- |
| **1. Goals and planning** |  |  |
| - 1. **Goal setting (behaviour)**   Set or agree on a goal defined in terms of the behaviour to be achieved  *Note: only code goal-setting if there is sufficient evidence that goal set as part of intervention;* *if goal unspecified or a behavioural outcome, code* ***1.3, Goal setting (outcome)****; if the goal defines a specific context, frequency, duration or intensity for the behaviour, also code* ***1.4, Action planning***  *Note: Code 1.1 for any discussion of a quit date, even if the specific date is not specified – due to the nature of the recordings.* | Agree on a quit date with the client  *Note: The specific quit date does not need to be contained within the transcript.* |  |
| **1.2 Problem solving**  Analyse, or prompt the person to analyse, factors influencing the behaviour and generate or select strategies that include overcoming barriers and/or increasing facilitators (includes ‘**Relapse Prevention***’ and ‘***Coping Planning***’*)  *Note: barrier identification without solutions is not sufficient. If the BCT does not include analysing the behavioural problem, consider* ***12.3****,* ***Avoidance/changing exposure to cues for the behaviour, 12.1, Restructuring the physical environment,*** ***12.2,*** ***Restructuring the social environment,*** *or* ***11.2, Reduce negative emotions*** | Identify specific triggers (e.g. being in a pub, feeling anxious) that generate the urge/want/need to smoke and develop strategies for avoiding environmental triggers that motivate smoking |  |
| **1.3 Goal setting (outcome)**  Set or agree on a goal defined in terms of a positive **outcome** of wanted behaviour  *Note:* *only code guidelines if set as a goal in an intervention context; if goal is a behaviour, code* ***1.1, Goal setting (behaviour)****; if goal unspecified code* ***1.3, Goal setting (outcome)*** | Set a goal with the client to have a CO-level of a non-smoker for the next meeting |  |
| **1.4 Action planning**  Prompt detailed planning of performance of the behaviour (must include at least one of context, frequency, duration and intensity). Context may be environmental (physical or social) or internal (physical, emotional or cognitive) (includes *‘***Implementation Intentions***’*)  *Note: evidence of action planning does not necessarily imply goal setting, only code latter if sufficient evidence* | Prompt planning of preparatory behaviour to facilitate quitting smoking (e.g. remove tobacco products from the house, tell others about the quit attempt) on specific days during the coming week  Prompt the client to make a plan how, when and where medication will be used. |  |
| **1.5 Review behaviour goals**  Review behaviour goal(s) jointly with the person and consider modifying goal(s) or behaviour change strategy in light of achievement. This may lead to re-setting the same goal, a small change in that goal or setting a new goal instead of (or in addition to) the first, or no change *Note:* *if goal specified in terms of behaviour, code* ***1.5, Review behaviour goal(s)****, if goal unspecified, code* ***1.7, Review outcome goal(s);*** *if* *discrepancy created consider also* ***1.6, Discrepancy between current behaviour and goal*** | Review with the client if the main goal of abstinence from cigarettes has been achieved. This may lead to resetting a quit date if the client has lapsed. |  |
| **1.6 Discrepancy between current behaviour and goal**  Draw attention to discrepancies between a person’s current behaviour (in terms of the *form, frequency, duration, or intensity* of that behaviour) and the person’s previously set outcome goals, behavioural goals or action plans (goes beyond self-monitoring of behaviour)  *Note: if discomfort is created only code* ***13.3, Incompatible beliefs*** *and not* ***1.6, Discrepancy between current behaviour and goal****;* *if goals are modified, also code* ***1.5, Review behaviour goal(s)*** *and/or* ***1.7, Review outcome goal(s)****; if feedback is provided, also code* ***2.2, Feedback on behaviour*** | Draw attention to the fact that the client has not met the goal of not smoking after the quit date. |  |
| **1.7 Review outcome goal**  Review outcome goal(s) jointly with the person and consider modifying goal(s) in light of achievement. This may lead to re-setting the same goal, a small change in that goal or setting a new goal instead of, or in addition to the first  *Note:* *if goal specified in terms of behaviour, code* ***1.5, Review behaviour goal(s)****, if goal unspecified, code* ***1.7, Review outcome goal(s);*** *if* *discrepancy created consider also* ***1.6, Discrepancy between current behaviour and goal*** | Review with the client whether the goal set for CO-reading has been met. This may lead to re-setting the same goal with regard to CO-levels or a small change in the goal on CO-levels. |  |
| **1.8 Behavioural contract**  Create a written specification of the behaviour to be performed, agreed on by the person, and witnessed by another  *Note:* *also code* ***1.1, Goal setting (behaviour)*** | Sign a written contract with the client stating that the client will not smoke after their quit date |  |
| **1.9 Commitment**  Ask the person to affirm or reaffirm statements indicating commitment to change the behaviour  *Note:* *if defined in terms of the behaviour to be achieved also code* ***1.1, Goal setting (behaviour)*** | Ask the person to use an “I will” statement to affirm or reaffirm a strong commitment (i.e. using the words “strongly”, “committed” or “high priority”) to quit smoking. |  |
| **2. Feedback and monitoring** | | |
| **2.1 Monitoring of behaviour by others without feedback**  Observe or record behaviour with the person’s knowledge as part of a behaviour change strategy  *Note: if monitoring is part of a data collection procedure rather than a strategy aimed at changing behaviour, do not code; if feedback given, code only* ***2.2, Feedback on behaviour****, and not* ***2.1, Monitoring of behaviour by others without feedback****; if monitoring outcome(s) code* ***2.5, Monitoring outcome(s) of behaviour by others without feedback****; if self-monitoring behaviour, code* ***2.3, Self-monitoring of behaviour*** | Ask a household member monitor how much the client smokes each day  Ask a pharmacist record if and how often smoking cessation medication is being picked up by the client |  |
| **2.2 Feedback on behaviour**  Monitor and provide informative or evaluative feedback on performance of the behaviour *(e.g. form, frequency, duration, intensity)*  *Note: if Biofeedback, code only* ***2.6, Biofeedback*** *and not* ***2.2, Feedback on behaviour****; if feedback is on* ***outcome(s)*** *of behaviour, code* ***2.7, Feedback on outcome(s) of behaviour****; if there is no clear evidence that feedback was given, code* ***2.1, Monitoring of behaviour by others without feedback****; if feedback on behaviour is evaluative e.g. praise, also code* ***10.4, Social reward*** | Give evaluative feedback on the number of cigarettes that the client smoked this week as measured in a daily diary  Give feedback indicating how well the client is adhering to stop smoking medication as reported in a daily diary |  |
| **2.3 Self-monitoring of behaviour**  Establish a method for the person to monitor and record their behaviour(s) as part of a behaviour change strategy *Note: if monitoring is part of a data collection procedure rather than a strategy aimed at changing behaviour, do not code; if monitoring of outcome of behaviour, code* ***2.4, Self-monitoring of outcome(s) of behaviour****;* *if monitoring is by someone else (without feedback), code* ***2.1, Monitoring of behaviour by others without feedback*** | Ask the client to record how many cigarettes they smoke each day in a diary  Ask the client to record daily medication adherence in a diary |  |
| **2.4 Self-monitoring of outcomes of behaviour**  Establish a method for the person to monitor and record the **outcome(s)** of their behaviour as part of a behaviour change strategy  *Note: if monitoring is part of a data collection procedure rather than a strategy aimed at changing behaviour, do not code; if monitoring behaviour, code* ***2.3, Self-monitoring of behaviour****; if monitoring is by someone else (without feedback), code* ***2.5, Monitoring outcome(s) of behaviour by others without feedback*** | Ask the client to write down each week of the smoking program how much money they have saved by not smoking |  |
| **2.5 Monitoring of outcome(s) behaviour by others without feedback**  Observe or record outcomes of behaviour with the person’s knowledge as part of a behaviour change strategy  *Note: if monitoring is part of a data collection procedure rather than a strategy aimed at changing behaviour, do not code; if feedback given, code only* ***2.7, Feedback on outcome(s) of behaviour****; if monitoring behaviour code* ***2.1, Monitoring of behaviour by others without feedback****; if self-monitoring outcome(s), code* ***2.4, Self-monitoring of outcome(s) of behaviour*** | Record expired-air carbon monoxide concentration to assess the extent of smoking exposure without giving feedback |  |
| **2.6 Biofeedback**  Provide feedback about the body *(e.g. physiological or biochemical state)* using an external monitoring device as part of a behaviour change strategy  *Note: if Biofeedback, code only* ***2.6, Biofeedback*** *and not* ***2.2, Feedback on behaviour*** *or* ***2.7, Feedback on outcome(s) of behaviour***  *Further note: if the measurement is taken with no feedback provided, code as* ***2.5, Monitoring of outcome(s) behaviour by others without feedback.***  *Note: Only code 2.6 when the feedback is provided to the client by the practitioner. Do not code for discussion of the CO monitor or instruction on how to use.* | Inform the person of their measured expired-air carbon monoxide concentration to encourage smoking cessation |  |
| **2.7 Feedback on outcome(s) of behaviour**  Monitor and provide feedback on the outcome of performance of the behaviour  *Note: if Biofeedback, code only* ***2.6, Biofeedback*** *and not* ***2.7, Feedback on outcome(s) of behaviour****; if feedback is on* ***behaviour*** *code* ***2.2, Feedback on behaviour****; if there is no clear evidence that feedback was given code* ***2.5, Monitoring outcome(s) of behaviour by others without feedback;*** *if feedback on behaviour is evaluative e.g. praise, also code* ***10.4, Social reward*** | Inform the client about the amount of money they have saved by not smoking during the past weeks |  |
| **3. Social support** | | |
| **3.1 Social support (unspecified)**  Advise on, arrange or provide social support *(e.g. from friends, relatives, colleagues,’ buddies’ or staff)* or non-contingent praise or reward for performance of the behaviour*.* It includes encouragement and counselling, but only when it is directed at the **behaviour**  *Note: attending a group class and/or mention of ‘follow-up’ does not necessarily apply this BCT, support must be explicitly mentioned; if practical, code* ***3.2, Social support (practical)****; if emotional, code* ***3.3, Social support (emotional)*** (includes ‘**Motivational interviewing**’ and **‘Cognitive Behavioural Therapy’**) | Advise the client to ask for social support during the quit attempt from family members, friends, or colleagues  Advise the person to call a ‘buddy’ when they experience an urge to smoke | ***A5 – Give options for additional and later support***  ***RC_10 – Provide reassurance***  ***15.1 – Verbal persuasion about capability*** |
| **3.2 Social support (practical)**  Advise on, arrange, or provide **practical** help *(e.g. from friends, relatives, colleagues, ‘buddies’ or staff)* for performance of the behaviour  *Note: if emotional, code* ***3.3, Social support (emotional)****; if general or unspecified, code* ***3.1, Social support (unspecified)*** *If only restructuring the physical environment or adding objects to the environment, code* ***12.1, Restructuring the physical environment*** *or* ***12.5, Adding objects to the environment;*** *attending a group or class and/or mention of ‘follow-up’ does not necessarily apply this BCT, support must be explicitly mentioned.* | Advise client to find someone to accompany them to a new non-smoking venue.  Advise client to ask partner to remove all smoking materials from their shared home.  Advise the client to get a friend, relative or colleague to drive them to the pharmacy to get stop smoking medication, if it is difficult to get to the pharmacy.  Ask the partner of the client to put their stop smoking medication on the breakfast tray so that the client remembers to take it. |  |
| **3.3 Social support (emotional)**  Advise on, arrange, or provide **emotional** social support *(e.g. from friends, relatives, colleagues, ‘buddies’ or staff)* for performance of the behaviour  *Note: if practical, code* ***3.2, Social support (practical)****; if unspecified, code* ***3.1, Social support (unspecified)*** | Advise the client to contact a ‘buddy’ to provide emotional support if the client has a difficult time staying abstinent. |  |
| **4. Shaping knowledge** | | |
| **4.1 Instruction how to perform the behaviour**  Advise or agree on how to perform the behaviour (includes ‘**Skills training**’)  *Note: when the person attends classes such as exercise or cookery, code* ***4.1, Instruction on how to perform the behaviour, 8.1, Behavioural practice/rehearsal*** *and* ***6.1, Demonstration of the behaviour*** | Advise that the most effective way of quitting is to stop abruptly rather than cut down gradually  Advise how to use an Nicotine Replacement Therapy (NRT) product (e.g. an inhaler) |  |
| **4.2 Information about antecedents**  Provide information about antecedents  (*e.g. social and environmental situations and events, emotions, cognitions)* that reliably predict performance of the behaviour | Provide information about the situations, events and emotions that have lead the client to starting smoking again  Advise the client to keep a record of situations in which they crave a cigarette | ***BM_10 – Explain the importance of abrupt cessation***  ***RC6 – Provide information on withdrawal symptoms*** |
| **4.3 Re-attribution**  Elicit perceived causes of behaviour and suggest alternative explanations *(e.g. external or internal and stable or unstable)* | If the person attributes their smoking to the need for stress reduction, suggest that the real cause may be nicotine dependence | ***RI7- Assess attitudes to smoking*** |
| **4.4 Behavioural experiments**  Advise on how to identify and test hypotheses about the behaviour, its causes and consequences, by collecting and interpreting data | If the client expects urges to smoke will become unbearable if they do not smoke, advise them to test this by waiting until urges dissipate when feeling the urge to smoke. |  |
| **4.5 Tell to act**  Tell the person to perform the target behaviour. This would normally involve an expectation of compliance, especially if given by someone with formal or informal authority. | Tell the client to stop smoking.  Advise client to take stop smoking medication. |  |
| **5. Natural consequences** | | |
| **5.1 Information about health consequences**  Provide information (e.g. written, verbal, visual) about health consequences of performing the behaviour  *Note: consequences can be for any target, not just the recipient(s) of the intervention; emphasising importance of consequences is not sufficient; if information about emotional consequences, code* ***5.6, Information about emotional consequences****; if about social, environmental or unspecified consequences code* ***5.3,*** ***Information about social and environmental consequences***  *Note: Code this BCT alongside 5.2 if the practitioner links the evidence (e.g., tar jar) to changes in health (good or bad) in respect of smoking/smoking cessation.* | Give the client a leaflet on the health consequences of smoking.  Explain verbally how the contents of a cigarette (e.g., tar) can cause health consequences (e.g., chest infections). |  |
| **5.2 Salience of consequences**  Use methods specifically designed to **emphasise** the consequences of performing the behaviour with the aim of making them more memorable (goes beyond informing about consequences)  *Note: if information about consequences, also code* ***5.1, Information about health consequences****,* ***5.6, Information about emotional consequences*** *or* ***5.3, Information about social and environmental consequences*** | Produce cigarette packets showing pictures of health consequences (e.g. diseased lungs), to highlight the dangers of continuing to smoke  Show the client a ‘tar jar’ which contains all tar that passes through the lungs in a year, to increase awareness of the health consequences of smoking |  |
| **5.3 Information about social and environmental consequences**  Provide information (e.g. written, verbal, visual) about social and environmental consequences of performing the behaviour  *Note: consequences can be for any target, not just the recipient(s) of the intervention; if information about health or consequences, code* ***5.1, Information about health consequences****; if about emotional consequences, code* ***5.6, Information about emotional consequences****; if unspecified, code* ***5.3, Information about social and environmental consequences*** | Provide information to the client about the negative health consequences of second-hand smoke to other people  Tell the client that cigarette butts are toxic to the natural environment |  |
| **5.4 Monitoring of emotional consequences**  Prompt assessment of **feelings** after attempts at performing the behaviour | Ask the client to record how they feel each morning after the quit attempt |  |
| **5.5 Anticipated regret**  Induce or raise awareness of expectations of future regret about performance of the unwanted behaviour  *Note: not including* ***5.6, Information about emotional consequences***; *if suggests adoption of a perspective or new perspective in order to change cognitions also code* ***13.2, Framing/reframing*** | Ask the client to assess the degree of regret they will feel if they do not quit smoking |  |
| **5.6 Information about emotional consequences**  Provide information (e.g. written, verbal, visual) about emotional consequences of performing the behaviour  *Note: consequences can be related to emotional health disorders (e.g. depression, anxiety) and/or states of mind (e.g. low mood, stress); not including* ***5.5, Anticipated regret****; consequences can be for any target, not just the recipient(s) of the intervention; if information about health consequences code* ***5.1, Information about health consequences****; if about social, environmental or unspecified code* ***5.3, Information about social and environmental consequences*** | Explain that quitting smoking increases happiness and life-satisfaction |  |
| **6. Comparison of behaviour** | | |
| **6.1 Demonstration of the behaviour**  Provide an observable sample of the performance of the behaviour, directly in person or indirectly e.g. via film, pictures, for the person to aspire to or imitate (includes ‘**Modelling**’). *Note:* if advised to practice, also code, ***8.1, Behavioural practice and rehearsal;*** *If provided with instructions on how to perform, also code* ***4.1, Instruction on how to perform the behaviour*** | Identify examples of celebrities who have quit smoking.  Demonstrate to the client how to use a specific Nicotine Replacement Therapy product (e.g. an inhaler) |  |
| **6.2 Social comparison**  Draw attention to others’ performance to allow comparison with the person’s own performance  *Note:* *being in a group setting does not necessarily mean that social comparison is actually taking place* | Show the proportion of patients who have had previous failed quit attempts and have successfully quit smoking  Draw attention to how well other group members have managed to use stop smoking medication and compare this with the person’s own performance |  |
| **6.3 Information about others’ approval**  Provide information about what other people think about the behaviour. The information clarifies whether others will like, approve or disapprove of what the person is doing or will do. | Tell the client that most people disapprove of smoking in public places  Explain that children are usually happy to hear that their parent is quitting smoking |  |
| **7. Associations** | | |
| **7.1 Prompts/cues**  Introduce or define environmental or social stimulus with the purpose of prompting or cueing the behaviour. The prompt or cue would normally occur at the time or place of performance  *Note: when a stimulus is linked to a specific action in an if-then plan including one or more of frequency, duration or intensity* *also code* ***1.4, Action planning****.* | Place some alternative to cigarettes e.g. sweets, where cigarettes would normally have been kept.  Send a text message every morning to remind the client to take stop smoking medication  Advise the client to put stop smoking medication next to a toothbrush to remind them to take it in the morning |  |
| **7.2 Cue signalling reward**  Identify an environmental stimulus that reliably predicts that reward will follow the behaviour (includes ***‘*Discriminative cue’**) | Advise that a financial incentive will be paid if the client is abstinent from smoking until delivery of the baby but not earlier |  |
| **7.3 Reduce prompts/cues**  Withdraw gradually prompts to perform the behaviour (includes ***‘*Fading*’***) | Gradually reduce the number of days on which the client records data such as craving or goal progression, which have been used to prompt smoking cessation.  Gradually reduce the number of reminders (e.g. text messages) the client receives to take their stop smoking medication |  |
| **7.4 Remove access to the reward**  Advise or arrange for the person to be separated from situations in which unwanted behaviour can be rewarded in order to reduce the behaviour (includes ***‘*Time out’**) | Advise client to remove all cigarettes from the house |  |
| **7.5 Remove aversive stimulus**  Advise or arrange for the removal of an aversive stimulus to facilitate behaviour change (includes ***‘*Escape learning*’***) | Arrange for the removal of something the client finds unpleasant (e.g. household chores) on adhering to stop smoking medication for a specified period of time |  |
| **7.6 Satiation**  Advise or arrange repeated exposure to a stimulus that reduces or extinguishes a drive for the unwanted behaviour | Advise a person to smoke much more than usual, to extinguish the urge to smoke |  |
| **7.7 Exposure**  Provide systematic confrontation with a feared stimulus to reduce the response to a later encounter | Agree a schedule by which a client, who fears they will start smoking again when visiting a pub, will systematically visit pubs to reduce this fear. |  |
| **7.8 Associative learning**  Present a neutral stimulus jointly with a stimulus that already elicits the behaviour repeatedly until the neutral stimulus elicits that behaviour (includes ***‘*Classical/Pavlovian Conditioning’**)  *Note: when a BCT involves reward or punishment, code one or more of:* ***10.2, Material reward (behaviour); 10.3, Non-specific reward; 10.4, Social reward, 10.9, Self-reward; 10.10, Reward (outcome)*** | When the client does not smoke when feeling sick, ask them to smoke rapidly, until they feel nauseous, so that smoking becomes associated with feeling sick. |  |
| **8. Repetition and substitution** | | |
| **8.1 Behavioural practice/rehearsal**  Prompt practice or rehearsal of the performance of the behaviour one or more times in a context or at a time when the performance may not be necessary, in order to increase habit and skill  *Note: if aiming to associate performance with the context, also code* ***8.3, Habit formation***  *Note: Code 8.1 if the practitioner advises or prompts the client to try/taste a sample of NRT.* | Advise to practice or rehearse not smoking in a context or time prior to the quit date, to increase the habit of not smoking.  Advise to try a sample of the nicotine gum before making a decision on which pharmacotherapy to use. |  |
| **8.2 Behaviour substitution**  Prompt substitution of the unwanted behaviour with a wanted or neutral behaviour  *Note: if this occurs regularly, also code* ***8.4, Habit reversal*** | Prompt the client to substitute smoking a cigarette with a wanted or neutral behaviour (e.g. brief physical exercise, eating a lollipop) |  |
| **8.3 Habit formation**  Prompt rehearsal and repetition of the behaviour in the same context repeatedly so that the context elicits the behaviour  *Note: also code* ***8.1, Behavioural practice/rehearsal*** | Advise to practice or rehearse not smoking in at the same context repeatedly, so that the context elicits abstinence |  |
| **8.4 Habit reversal**  Prompt rehearsal and repetition of an alternative behaviour to **replace** an unwanted habitual behaviour *Note: also code* ***8.2, Behaviour substitution*** | Prompt repetition of a wanted or neutral behaviour (e.g. brief physical exercise, eating a lollipop) at the time or places where the client would previously have smoked cigarettes |  |
| **8.5 Generalisation of target behaviour**  Advise to perform the wanted behaviour, which is already performed in a particular situation, in another situation | Advise that since people already do not smoke in certain situations (e.g., on during travel in public transport, during dinner) to extend these non-smoking situations to another situation in which they usually smoke |  |
| **8.6 Graded tasks**  Set easy-to-perform tasks, making them increasingly difficult, but achievable, until behaviour is performed | Ask the client to get through a morning without smoking, then to get through a whole day without smoking until one has achieved a larger target of not smoking for one week |  |
| **9. Comparison of outcomes** | | |
| **9.1 Credible source**  Present verbal or visual communication from a credible source **in favour of or against the behaviour** *Note:* *code this BCT if source generally agreed on as credible e.g., health professionals, celebrities or words used to indicate expertise or leader in field and if the communication has the aim of persuading; if information about health consequences, also code* ***5.1, Information about health consequences****, if about emotional consequences, also code* ***5.6, Information about emotional consequences****; if about social, environmental or unspecified consequences also code* ***5.3, Information about social and environmental consequences*** | Present verbal or visual communication from a high-status health expert in favour of smoking cessation |  |
| **9.2 Pros and cons**  Advise the person to identify and compare reasons for wanting (pros) and not wanting to (cons) change the behaviour (includes ‘**Decisional balance’***)*  *Note:* *if providing information about health consequences, also code* ***5.1, Information about health consequences****; if providing information about emotional consequences, also code* ***5.6, Information about emotional consequences****; if providing information about social, environmental or unspecified consequences also code* ***5.3, Information about social and environmental consequences*** | Advise the client to generate a list of reasons why they do and why they do not want to stop smoking |  |
| **9.3 Comparative imagining of future outcomes**  Prompt or advise the imagining and comparing of future outcomes of changed versus unchanged behaviour | Prompt the client to imagine the possible future in which they have stopped smoking and compare it with a future where they continue to smoke. |  |
| **10. Reward and threat** | | |
| **10.1 Material incentive (behaviour)**  Inform that money, vouchers or other valued objects ***will be*** delivered if and only if there has been effort and/or progress in performing the behaviour (includes ***‘*Positive reinforcement’**)  *Note: if incentive is social, code* ***10.5, Social incentive*** *if unspecified code* ***10.6,*** ***Non-specific incentive*** *and not* ***10.1, Material incentive (behaviour)*** *if incentive is for* ***outcome,*** *code* ***10.8, Incentive (outcome).*** *If reward is delivered also code one of:* ***10.2, Material reward (behaviour); 10.3, Non-specific reward; 10.4, Social reward, 10.9, Self-reward; 10.10, Reward (outcome)*** | Inform that a financial payment will be made each month in pregnancy that the woman has not smoked  Inform the client that vouchers will be given for each week in which stop smoking medication is used correctly |  |
| **10.2 Material reward (behaviour)**  Arrange for the delivery of money, vouchers or other valued objects if and only if there ***has been*** effort and/or progress in performing the behaviour (includes ‘**Positive reinforcement’**)  *Note: If reward is social, code* ***10.4, Social reward****, if unspecified code* ***10.3, Non-specific reward****, and not* ***10.1, Material reward (behaviour)****, if reward is for* ***outcome****, code* ***10.10, Reward (outcome).*** *If informed of reward in advance of rewarded behaviour, also code one of:* ***10.1, Material incentive (behaviour); 10.5, Social incentive; 10.6, Non-specific incentive; 10.7, Self-incentive; 10.8, Incentive (outcome)*** | Arrange for the person to receive money that would have been spent on cigarettes if and only if the smoker has not smoked for one month |  |
| **10.3 Non-specific reward**  Arrange delivery of a reward if and only if there ***has been*** effort and/or progress in performing the behaviour (includes ‘**Positive reinforcement’**)  *Note: if reward is material, code* ***10.2, Material reward (behaviour)****, if social, code* ***10.4, Social reward****, and not* ***10.3, Non-specific reward****, if reward is for* ***outcome*** *code* ***10.10, Reward (outcome).*** *If informed of reward in advance of rewarded behaviour, also code one of****: 10.1, Material incentive (behaviour); 10.5, Social incentive; 10.6, Non-specific incentive; 10.7, Self-incentive; 10.8, Incentive (outcome)*** | Identify something (e.g. an activity such as a visit to the cinema) that the person values and arrange for this to be delivered if and only if they have stayed abstinent for a week |  |
| **10.4 Social reward (behaviour)**  Arrange verbal or non-verbal reward if and only if there ***has been*** effort and/or progress in performing the behaviour (includes ‘**Positive reinforcement**’)  *Note: if reward is material, code* ***10.2, Material reward (behaviour)****, if unspecified code* ***10.3, Non-specific reward****, and not* ***10.4, Social reward****, if reward is for* ***outcome*** *code* ***10.10, Reward (outcome).*** *If informed of reward in advance of rewarded behaviour, also code one of****: 10.1, Material incentive (behaviour); 10.5, Social incentive; 10.6, Non-specific incentive; 10.7, Self-incentive; 10.8, Incentive (outcome)*** | Congratulate the person for each week they do not smoke  Give praise or encouragement for improving their adherence to stop smoking medication |  |
| **10.5 Social incentive**  Inform that a verbal or non-verbal reward ***will be*** delivered if and only if there has been effort and/or progress in performing the behaviour (includes ‘**Positive reinforcement’**)  *Note: if incentive is material, code* ***10.1, Material incentive (behaviour)****, if unspecified code* ***10.6, Non-specific incentive****, and not* ***10.5, Social incentive****, if incentive is for* ***outcome*** *code* ***10.8, Incentive (outcome).*** *If reward is delivered also code one of****: 10.2, Material reward (behaviour); 10.3, Non-specific reward; 10.4, Social reward, 10.9, Self-reward; 10.10, Reward (outcome)*** | Inform the client that they will be congratulated by their partner each week they do not smoke |  |
| **10.6 Non-specific incentive**  Inform that a reward ***will be*** delivered if and only if there has been effort and/or progress in performing the behaviour (includes ‘**Positive reinforcement’**)  *Note: if incentive is material, code* ***10.1, Material incentive (behaviour)****, if social, code* ***10.5, Social incentive*** *and not* ***10.6, Non-specific incentive****; if incentive is for* ***outcome*** *code* ***10.8, Incentive (outcome).*** *If reward is delivered also code one of****: 10.2, Material reward (behaviour); 10.3, Non-specific reward; 10.4, Social reward, 10.9, Self-reward; 10.10, Reward (outcome)*** | Inform the client that something that they value (not money, e.g. an activity such as a visit to the cinema) will be given if and only if they have not smoked for a month |  |
| **10.7 Self-incentive**  Plan to reward self in future if and only if there has been effort and/or progress in performing the behaviour  *Note: if self-reward is material, also code* ***10.1, Material incentive (behaviour)****, if social, also code* ***10.5, Social incentive****, if unspecified, also code* ***10.6, Non-specific incentive****; if incentive is for* ***outcome*** *code* ***10.8, Incentive (outcome).*** *If reward is delivered also code one of:* ***10.2, Material reward (behaviour); 10.3, Non-specific reward; 10.4, Social reward, 10.9, Self-reward; 10.10, Reward (outcome)*** | Ask the client to make a plan to give them self a reward (e.g. nice clothes) if and only if they have not smoked for 2 weeks |  |
| **10.8 Incentive (outcome)**  Inform that a reward ***will be*** delivered if and only if there has been effort and/or progress in achieving the behavioural **outcome** (*includes* ***‘*Positive reinforcement*’***)  *Note: this includes social, material, self- and non-specific incentives for outcome; if incentive is for the* ***behaviour*** *code* ***10.5****,* ***Social*** ***incentive****,* ***10.1, Material*** ***incentive (behaviour)****,* ***10.6, Non****-****specific incentive*** *or* ***10.7****,* ***Self****-****incentive*** *and not* ***10.8, Incentive (outcome).*** *If reward is delivered also code one of:* ***10.2, Material reward (behaviour); 10.3, Non-specific reward; 10.4, Social reward, 10.9, Self-reward; 10.10, Reward (outcome)*** | Inform the client that a reward will be delivered if and only if they have made progress in reducing CO-levels of expired air. |  |
| **10.9 Self-reward**  Prompt self-praise or self-reward if and only if there ***has been*** effort and/or progress in performing the behaviour  *Note: if self-reward is material, also code* ***10.2, Material reward (behaviour)****, if social, also code* ***10.4, Social reward****, if unspecified, also code* ***10.3, Non-specific reward****; if reward is for* ***outcome*** *code* ***10.10, Reward (outcome).*** *If informed of reward in advance of rewarded behaviour, also code one of:* ***10.1, Material incentive (behaviour); 10.5, Social incentive; 10.6, Non-specific incentive; 10.7, Self-incentive; 10.8, Incentive (outcome)*** | Encourage the client to praise themselves if and only if they have been abstinent for 2 days |  |
| **10_10 Reward (outcome)**  Arrange for the delivery of a reward if and only if there ***has been*** effort and/or progress in achieving the behavioural **outcome** (includes ‘**Positive reinforcement**’)  *Note: this includes social, material, self- and non-specific rewards for outcome; if reward is for the* ***behaviour*** *code* ***10.4****,* ***Social*** ***reward****,* ***10.2, Material*** ***reward (behaviour)****,* ***10.3,*** ***Non****-****specific*** ***reward*** *or* ***10.9****,* ***Self****-****reward*** *and not* ***10.10, Reward (outcome).*** *If informed of reward in advance of rewarded behaviour, also code one of****: 10.1, Material incentive (behaviour); 10.5, Social incentive; 10.6, Non-specific incentive; 10.7, Self-incentive; 10.8, Incentive (outcome)*** | Arrange for the client to save all the money they would have spent on cigarettes and buy something they value with it (e.g. a book) if and only if they have received low readings on a carbon monoxide reader for a month |  |
| **10_11 Future punishment**  Inform that future punishment or removal of reward will be a consequence of performance of an unwanted behaviour (may include fear arousal) (includes ***‘*Threat*’***) | Inform the client that smoking at work is likely to result in loss of employment with the NHS |  |
| **11. Regulation** | | |
| **11.1 Pharmacological support**  Provide information, or encourage the use of or adherence to, drugs to facilitate behaviour change  *Note: if pharmacological support to reduce negative emotions (i.e. anxiety) then also code* ***11.2, Reduce negative emotions*** | Enact the necessary procedures to ensure that the client gets their stop smoking medication easily and without charge where appropriate |  |
| **11.2 Reduce negative emotions**  Advise on ways of reducing negative emotions to facilitate performance of the behaviour (includes ‘**Stress Management**’)  *Note: if includes analysing the behavioural problem, also code* ***1.2****,* ***Problem solving*** | Advise on the use of specific relaxation techniques to the client to make it easier to stay abstinent |  |
| **11.3 Conserving mental resources**  Advise on ways of minimising demands on mental resources to facilitate behaviour change | Advise the client to relax as much as possible and get a good night’s sleep while trying to stop smoking. |  |
| **11.4 Paradoxical instructions**  Advise to engage in some form of the unwanted behaviour with the aim of reducing motivation to engage in that behaviour | Advise the client to smoke twice as many cigarettes as they usually do to reduce the motivation to smoke |  |
| **12. Antecedents** | | |
| **12.1 Restructuring the physical environment**  Change, or advise to change the **physical** environment in order to facilitate performance of the wanted behaviour or create barriers to the unwanted behaviour (other than prompts/cues, rewards and punishments)  *Note: this may also involve* ***12.3, Avoidance/reducing exposure to cues for the behaviour****;* *if restructuring of the social environment code* ***12.2, Restructuring the social environment,***  *if only adding objects to the environment, code* ***12.5, Adding objects to the environment*** | Advise the client to remove all tobacco products, lighters and ashtrays from their surroundings |  |
| **12.2 Restructuring the social environment**  Change, or advise to change the **social** environment in order to facilitate performance of the wanted behaviour or create barriers to the unwanted behaviour (other than prompts/cues, rewards and punishments)  *Note: this may also involve* ***12.3, Avoidance/reducing exposure to cues for the behaviour****; if also restructuring of the physical environment also code* ***12.1, Restructuring the physical environment*** | Advise the client to spend less time with friends who smoke.  Advise the client to ask smoking family members, housemates, friends and/or colleagues not to smoke in their presence |  |
| **12.3 Avoidance/reducing exposure to cues for the behaviour**  Advise on how to avoid exposure to specific social and contextual/physical cues for the behaviour, including changing daily or weekly routines  *Note:* *this may also involve* ***12.1, Restructuring the physical environment*** and/or ***12.2, Restructuring the social environment***; if the BCT includes analysing the behavioural problem, only code ***1.2*,** ***Problem solving*** | Advise the client to avoid situations such as pubs in which common triggers to smoke occur by changing daily/weekly routines |  |
| **12.4 Distraction**  Advise or arrange to use an alternative focus for attention to avoid triggers for the unwanted behaviour | Suggest to a person who is trying to quit smoking to focus on a topic they enjoy (e.g. holiday plans) when they feel the urge to smoke |  |
| **12.5 Adding objects to the environment**  Add objects to the environment in order to facilitate performance of the behaviour  *Note: Provision of information (e.g. written, verbal, visual) in a booklet or leaflet is insufficient. If this is accompanied by social support, also code* ***3.2, Social support (practical)****; if the environment is changed beyond the addition of objects, also code* ***12.1, Restructuring the physical environment*** | Arrange for cigarette packets to carry anti-smoking messages and images to encourage reduced smoking rates  Give the client an electronic medication monitoring device, which indicates whether or not they have taken their medication today. |  |
| **13. Identity** | | |
| **13.1 Identification of self as a role-model**  Inform that one's own behaviour may be an example to others | Inform the client that if they quit smoking they would be a good role-model for smoking friends |  |
| **13.2 Framing/reframing**  Suggest the deliberate adoption of a perspective or new perspective on behaviour (e.g. its purpose) in order to change cognitions or emotions about performing the behaviour (includes ‘**Cognitive structuring**’); *If information about consequences then code* ***5.1, Information about health consequences, 5.6, Information about emotional consequences*** *or* ***5.3, Information about social and environmental consequences*** *instead of* ***13.2, Framing/reframing*** | Suggest the client deliberately adopts a different, more positive view on past failed quit attempts or lapses (e.g. advise that lapses can be viewed as a valuable learning experience that can help with this quit attempt) |  |
| **13.3 Incompatible beliefs**  Draw attention to discrepancies between current or past behaviour and self-image, in order to create discomfort (includes ***‘*Cognitive dissonance’**) | Draw attention to the fact that the client sees themselves as athletic, but at the same time is reducing their athletic ability by smoking |  |
| **13.4 Valued self-identity**  Advise the person to write or complete rating scales about a cherished value or personal strength as a means of affirming the person’s identity as part of a behaviour change strategy (includes ***‘*Self-affirmation’**) | Advise the client to write or talk about a cherished value or personal strength as a means to affirming their identity in order to strengthen their ability to quit smoking |  |
| ***13.5 Identity associated with changed behaviour***  Advise the person to construct a new self-identity as someone who ‘used to engage with the unwanted behaviour’  *Note: Code 13.5 if the practitioners actively promotes the client to think like a non-smoker. Do not code if the practitioner merely asks, “How much do you agree that I am able to see myself as a non-smoker?”* | Ask the person to articulate their new identity as an ‘ex-smoker’ |  |
| **14. Scheduled consequences** | | |
| **14.1 Behaviour cost**  Arrange for withdrawal of something valued if and only if an unwanted behaviour is performed (includes ‘**Response cost’**). Note if withdrawal of contingent reward code*,* ***14.3, Remove reward*** | Subtract money from a prepaid refundable deposit when a cigarette is smoked |  |
| **14.2 Punishment**  Arrange for aversive consequence contingent on the performance of the unwanted behaviour | Arrange that the client has to do their housemates tasks (e.g. the laundry) when they smoke a cigarette this week |  |
| **14.3 Remove reward**  Arrange for discontinuation of contingent reward following performance of the unwanted behaviour (includes **‘Extinction’**) | Arrange for household members to ignore the client if the client is smoking a cigarette |  |
| **14.4 Reward approximation**  Arrange for reward following any approximation to the target behaviour, gradually rewarding only performance closer to the wanted behaviour (includes ***‘*Shaping*’***) | Arrange for money to be given to the client each week they successfully cut down smoking by any number of cigarettes gradually requiring this reduction in number of cigarettes to be greater and approach cessation. |  |
| **14.5 Rewarding completion**  Build up behaviour by arranging reward following final component of the behaviour; gradually add the components of the behaviour that occur earlier in the behavioural sequence (includes ***‘*Backward chaining’**)  *Note: also code one of* ***10.2, Material reward (behaviour); 10.3, Non-specific reward; 10.4, Social reward, 10.9, Self-reward; 10.10, Reward (outcome)*** | Reward quitting in pregnant smokers; then make reward contingent on staying abstinent for a week; then make reward contingent on staying abstinent for a month; then make reward contingent on staying abstinent until the baby is born.  Reward taking supplied stop smoking medication; then make reward contingent on prompted purchasing and taking medication; then make reward contingent on self-initiated purchasing of medication and taking medication. |  |
| **14.6 Situation-specific reward**  Arrange for reward following the behaviour in one situation but not in another (includes ***‘*Discrimination training’**)  *Note:* *also code one of* ***10.2, Material reward (behaviour); 10.3, Non-specific reward; 10.4, Social reward, 10.9, Self-reward; 10.10, Reward (outcome)*** | Arrange for a reward to be given to the client for not smoking after a meal but do not give a reward for not smoking during a meal (when they would normally not smoke). |  |
| **14.7 Reward incompatible behaviour**  Arrange reward for responding in a manner that is incompatible with a previous response to that situation (includes ***‘*Counter-conditioning’**)  *Note: also code one of* ***10.2, Material reward (behaviour); 10.3, Non-specific reward; 10.4, Social reward, 10.9, Self-reward; 10.10, Reward (outcome)*** | Arrange for client to be rewarded for arranging to meet friends, whom they have previously met in a venue where smoking was allowed, in a non-smoking venue |  |
| **14.8 Reward alternative behaviour**  Arrange reward for performance of an alternative to the unwanted behaviour (includes ***‘*Differential reinforcement*’***)  *Note: also code one of* ***10.2, Material reward (behaviour); 10.3, Non-specific reward; 10.4, Social reward, 10.9, Self-reward; 10.10, Reward (outcome);*** *consider also coding* ***1.2, Problem solving*** | Arrange for the client to receive a reward if he eats a lollipop when feeling the urge to smoke rather than smoking a cigarette.  Arrange for the client to receive a reward if they buy something else (e.g. a magazine) at their local cigarette shop, rather than cigarettes. |  |
| **14.9 Reduce reward frequency**  Arrange for rewards to be made contingent on increasing duration or frequency of the behaviour (includes ***‘*Thinning*’***)  *Note: also code one of* ***10.2, Material reward (behaviour); 10.3, Non-specific reward; 10.4, Social reward, 10.9, Self-reward; 10.10, Reward (outcome)*** | Arrange reward for each day without smoking, then for each week, then for each month, then every two months and so on |  |
| **14_10 Remove punishment**  Arrange for removal of an unpleasant consequence contingent on performance of the wanted behaviour (includes ***‘*Negative reinforcement’**) | Arrange that a housemate will take up some of the disliked household tasks (e.g. cleaning) if the client does not smoke for a week |  |
| **15. Self-belief** | | |
| **15.1 Verbal persuasion about capability**  Tell the person that they can successfully perform the wanted behaviour, arguing against self-doubts and asserting that they can and will succeed | Tell the person that they can quit smoking even though previous quit attempts have not been successful | ***RC_10 – Provide reassurance***  ***3.1 – Social support (unspecified)*** |
| **15.2 Mental rehearsal of successful performance**  Advise to practise imagining performing the behaviour successfully in relevant contexts | Advise the client to imagine successfully not smoking in specific situations in which one usually smokes (e.g. after dinner, at a party). |  |
| **15.3 Focus on past success**  Advise to think about or list previous successes in performing the behaviour (or parts of it) | Ask the client to describe times when they have successfully gone without a cigarette in a situation in which they would normally have smoked |  |
| **15.4 Self-talk**  Prompt positive self-talk (aloud or silently) before and during the behaviour | Prompt the client to tell themselves when they feel urges to smoke that they will be able to get through the day without smoking  Prompt a person to tell themselves that using stop smoking medication will make them feel better because it reduces withdrawal symptoms |  |
| **16. Covert learning** | | |
| **16.1 Imaginary punishment**  Advise to imagine performing the **unwanted** behaviour in a real-life situation followed by imagining an unpleasant consequence (includes ***‘*Covert sensitisation’**) | Advise the client to imagine starting smoking again and feeling unhealthy, unhappy and low on energy |  |
| **16.2 Imaginary reward**  Advise to imagine performing the **wanted** behaviour in a real-life situation followed by imagining a pleasant consequence (includes ***‘*Covert conditioning’**) | Advise the client to imagine feeling healthier, happier and more energetic after successfully having quit smoking |  |
| **16.3 Vicarious consequences**  Prompt observation of the consequences (including rewards and punishments) for others when they perform the behaviour  *Note:* *if observation of health consequences, also code* ***5.1, Information about health consequences****; if of emotional consequences, also code* ***5.6, Information about emotional consequences****, if of social, environmental or unspecified consequences, also code* ***5.3,*** ***Information about social and environmental consequences*** | Draw attention to compliments other people get when they quit smoking |  |

**Supplementary material S2.**

Multilevel modelling results for the association between specific BCTs delivered and 2-week point prevalence smoking abstinence at 4-week follow-up, 8-week follow-up and continued abstinence at 6-month follow-up

| **BCTs delivered** | **Estimate** | **8-Week (2-Week PP) Abstinence**  ***p (*95% *CI)*** | **Estimate** | **4-Week (2-Week PP) Abstinence**  ***p (*95 % *CI)*** | **Estimate** | **6-Month Continued Abstinence**  ***p (*95% *CI)*** |
| --- | --- | --- | --- | --- | --- | --- |
| **BM10** Explain the importance of abrupt cessation | .35 | .45 (-1.21 to 1.91) | -.71 | .25 (-0.19 to 0.52) | -.42 | .37 (-1.34 to 0.50) |
| **BM13** Create or reinforce negative associations | .21 | .17 (-0.10 to 0.52) | .07 | .52 (-0.14 to 0.29) | -.09 | .24 (-0.25 to 0.06) |
| **BS13** Advise on methods of weight control | .02 | .96 (-0.65 to 0.68) | .05 | .83 (-0.42 to 0.52) | -.52 | .02 (-0.86 to -0.19)*^a^ |
| **A4** Ask about experiences of stop smoking medication that the smoker is using | -.14 | .89 (-2.38 to 2.09) | -.93 | .16 (-2.22 to 0.36) | -.07 | .89 (-1.20 to 1.05) |
| **A5** Give options for additional and later support | -.37 | .21 (-0.97 to 0.22) | -.30 | .15 (-0.70 to 0.10) | -.26 | .08 (-0.55 to 0.03) |
| **RD1** Tailor interactions appropriately | -.27 | .07 (-0.55 to 0.02) | .03 | .81 (-0.19 to 0.24) | -.03 | .75 (-0.19 to 0.14) |
| **RD2** Emphasise choice | -.14 | .29 (-0.42 to 0.13) | -.04 | .68 (-0.24 to 0.16) | -.07 | .34 (-0.22 to 0.08) |
| **RD3** Promote engagement with the programme | -.05 | .91 (-0.95 to 0.85) | .57 | .08 (-0.06 to 1.21) | -.20 | .39 (-0.66 to 0.16) |
| **RI1** Assess current and past smoking behavior | .03 | .86 (-0.30 to 0.36) | .15 | .21 (-0.08 to 0.38) | -.02 | .78 (-0.21 to 0.16) |
| **RI2** Assess current readiness and ability to quit | -.17 | .17 (-0.41 to 0.08) | .02 | .84 (-0.15 to 0.19) | -.10 | .11 (-0.22 to 0.02) |
| **RI3** Assess past history of quit attempts | -.06 | .66 (-0.35 to 0.23) | -.07 | .47 (-0.27 to 0.13) | .06 | .40 (-0.09 to 0.22) |
| **RI4** Assess withdrawal symptoms | -.71 | .14 (-1.69 to 0.26) | .45 | .16 (-0.17 to 1.06) | -.39 | .08 (-0.84 to 0.05) |
| **RI5** Assess nicotine dependence | -.04 | .71 (-0.23 to 0.16) | .10 | .16 (-0.04 to 0.24) | -.04 | .48 (-0.15 to 0.07) |
| **RI6** Assess number of contacts who smoke | .15 | .28 (-0.13 to 0.43) | -.009 | .93 (-0.21 to 0.19) | -.05 | .51 (-0.20 to 0.10) |
| **RI7** Assess attitudes to smoking | -.16 | .13 (-0.38 to 0.05) | .03 | .72 (-0.13 to 0.19) | .005 | .94 (-0.12 to 0.13) |
| **RI8** Assess level of social support | .003 | .99 (-0.38 to 0.39) | .15 | .28 (-0.12 to 0.42) | .12 | .24 (-0.08 to 0.33) |
| **RI9** Explain how tobacco dependence develops | .24 | .23 (-0.16 to 0.65) | .05 | .72 (-0.24 to 0.35) | .09 | .41 (-0.14 to 0.32) |
| **RI10** Assess physiological and mental functioning | -.16 | .20 (-0.42 to 0.10) | .04 | .70 (-0.15 to 0.23) | -.07 | .36 (-0.21 to 0.08) |
| **RC1** Build general rapport | -.17 | .48 (-0.64 to 0.31) | -.09 | .62 (-0.46 to 0.27) | -.17 | .21 (-0.43 to 0.10) |
| **RC3** Explain the purpose of CO monitoring | -.03 | .71 (-0.22 to 0.15) | .17 | .61 (-0.01 to 0.34) | .002 | .97 (-0.12 to 0.13) |
| **RC4** Explain expectations regarding the treatment programme | -.005 | .98 (-0.36 to 0.35) | .06 | .67 (-0.20 to 0.31) | -.005 | .96 (-0.20 to 0.19) |
| **RC5** Offer/direct towards appropriate written materials | -.18 | .10 (-0.40 to 0.04) | -.04 | .62 (-0.20 to 0.12) | -.09 | .11 (-0.21 to 0.02) |
| **RC6** Provide information on withdrawal symptoms | -.14 | .23 (-0.37 to 0.10) | -.01 | .90 (-0.17 to 0.15) | -.06 | .35 (-0.18 to 0.07) |
| **RC7** Use reflective listening | .45 | .05 (0.001 to 0.91)* | -.01 | .95 (-0.35 to 0.33) | .04 | .74 (-0.23 to 0.31) |
| **RC8** Elicit client views | -.04 | .78 (-0.37 to 0.28) | -.15 | .20 (-0.7 to 0.08) | .02 | .79 (-0.15 to 0.20) |
| **RC9** Summarise information/confirm client decisions | -.09 | .52 (-0.38 to 0.20) | .16 | .10 (-0.03 to 0.34) | -.06 | .37 (-0.20 to 0.07) |
| **RC10** Provide reassurance | .49 | .25 (-0.36 to 1.34) | .53 | .09 (-0.09 to 1.14) | .03 | .90 (-0.45 to 0.51) |
| **OTH1** Schedule follow-up appointment | .18 | .07 (-0.02 to 0.38) | -.004 | .96 (-0.16 to 0.15) | -.001 | .98 (-0.12 to 0.12) |
| **OTH2** Physiological effects of nicotine/smoking | -.04 | .77 (-0.30 to 0.22) | -.02 | .82 (-0.20 to 0.16) | -.10 | .12 (-0.23 to 0.03) |
| **1.1** Goal setting (behavior) | .33 | .20 (-0.18 to 0.84) | 0.20 | .32 (-0.19 to 0.59) | .01 | .95 (-0.29 to 0.31) |
| **1.2** Problem solving | -.15 | .22 (-0.40 to 0.10) | -.02 | .86 (-0.20 to 0.17) | -.01 | .87 (-0.15 to 0.13) |
| **1.3** Goal setting (outcome) | -.04 | .82 (-0.44 to 0.35) | .18 | .21 (-0.10 to 0.46) | -.04 | .71 (-0.26 to 0.18) |
| **1.4** Action planning | .004 | .98 (-0.36 to 0.37) | -.05 | .73 (-0.31 to 0.22) | -.13 | .20 (-0.33 to 0.07) |
| **1.5** Review behavior goals | - | - | - | - | - | - |
| **1.6** Discrepancy between current behavior and goal | - | - | - | - | - | - |
| **1.7** Review outcome goal | .60 | .70 (-2.55 to 3.74) | 1.02 | .37 (-1.20 to 3.24) | 1.14 | .17 (-0.47 to 2.74) |
| **1.8** Behavioral contract | - | - | - | - | - | - |
| **1.9** Commitment | -2.03 | .32 (-6.11 to 2.05) | -2.32 | .14 (-5.37 to 0.74) | -1.79 | .12 (-4.09 to 0.51) |
| **2.1** Monitoring of behavior by others without feedback | - | - | - | - | - | - |
| **2.2** Feedback on behavior | -.36 | .08 (-0.77 to 0.05) | .20 | .11 (-0.05 to 0.45) | -.11 | .23 (-0.29 to 0.07) |
| **2.3** Self-monitoring of behavior | -1.04 | .05 (-2.11 to 0.02) | .43 | .19 (-0.21 to 1.07) | -.34 | .15 (-0.80 to 0.13) |
| **2.4** Self-monitoring of outcomes of behavior | -.14 | .44 (-0.53 to 0.24) | .004 | .98 (-0.25 to 0.25) | -.05 | .57 (-0.26 to 0.15) |
| **2.5** Monitoring of outcome(s) behavior without feedback | - | - | - | - | - | - |
| **2.6** Biofeedback | .32 | .08 (-0.04 to 0.67) | .02 | .88 (-0.26 to 0.30) | .02 | .80 (-0.19 to 0.23) |
| **2.7** Feedback on outcome(s) of behavior | .60 | .70 (-2.55 to 3.74) | 1.02 | .37 (-1.20 to 3.24) | 1.14 | .17 (-0.47 to 2.74) |
| **3.1** Social support (unspecified) | -.21 | .11 (-0.46 to 0.05) | .11 | .24 (-0.08 to 0.31) | .02 | .83 (-0.13 to 0.16) |
| **3.2** Social support (practical) | .66 | .41 (-0.98 to 2.30) | -.81 | .15 (-1.91 to 0.30) | -.19 | .67 (-1.09 to 0.71) |
| **3.3** Social support (emotional) | .15 | .77 (-0.90 to 1.21) | -.13 | .73 (-0.88 to 0.61) | .25 | .38 (-0.32 to 0.81) |
| **4.1** Instruction how to perform the behavior | -.12 | .39 (-0.39 to 0.16) | .13 | .22 (-0.08 to 0.35) | .05 | .57 (-0.11 to 0.21) |
| **4.2** Information about antecedents | .14 | .43 (-0.21 to 0.49) | -.001 | 1.00 (-0.27 to 0.27) | .14 | .14 (-0.05 to 0.34) |
| **4.3** Re-attribution | -.27 | .31 (-0.80 to 0.26) | -.16 | .43 (-0.56 to 0.24) | -.16 | .30 (-0.46 to 0.14) |
| **4.4** Behavioral experiments | .95 | .52 (-2.15 to 4.04) | .15 | .87 (-1.71 to 2.01) | -1.60 | .02 (-2.93 to -0.26)*^a^ |
| **4.5** Tell to act | -.31 | .45 (-1.16 to 0.53 | -.27 | .39 (-0.89 to 0.35) | -.45 | .06 (-0.92 to 0.01) |
| **5.1** Information about health consequences | -.08 | .39 (-0.28 to 0.11) | -.02 | .81 (-0.16 to 0.12) | -.10 | .06 (-0.20 to 0.002) |
| **5.2** Salience of consequences | -.08 | .60 (-0.37 to 0.22) | .002 | .98 (-0.19 to 0.20) | -.10 | .18 (-0.24 to 0.04) |
| **5.3** Information about social and environmental consequences | -.02 | .91 (-0.44 to 0.39) | -.02 | .91 (-0.32 to 0.29) | -.13 | .28 (-0.36 to 0.12) |
| **5.4** Monitoring of emotional consequences | -.002 | .00 (-1.04 to 1.03) | -.18 | .63 (-0.93 to 0.56) | .18 | .54 (-0.39 to 0.75) |
| **5.5** Anticipated regret | .95 | .25 (-0.70 to 2.60) | -.38 | .58 (-1.74 to 0.97) | .10 | .84 (-0.90 to 1.11) |
| **5.6** Information about emotional consequences | -.38 | .19 (-0.97 to 0.21) | -.03 | .89 (-0.41 to 0.36) | -.38 | .01 (-0.65 to -0.10)*^a^ |
| **6.1** Demonstration of the behavior | -.32 | .30 (-0.94 to 0.30) | -.22 | .34 (-0.68 to 0.24) | -.13 | .46 (-0.50 to 0.23) |
| **6.2** Social comparison | - | - | - | - | - | - |
| **6.3** Information about others’ approval | - | - | - | - | - | - |
| **7.1** Prompts/cues | -.31 | .07 (-0.65 to 0.03) | .19 | .13 (-0.06 to 0.44) | -.05 | .59 (-0.25 to 0.15) |
| **7.2** Cue signalling reward | - | - | - | - | - | - |
| **7.3** Reduce prompts/cues | - | - | - | - | - | - |
| **7.4** Remove access to the reward | - | - | - | - | - | - |
| **7.5** Remove aversive stimulus | - | - | - | - | - | - |
| **7.6** Satiation | - | - | - | - | - | - |
| **7.7** Exposure | - | - | - | - | - | - |
| **7.8** Associative learning | - | - | - | - | - | - |
| **8.1** Behavioral practice/rehearsal | .28 | .61 (-0.85 to 1.41) | -.15 | .69 (-0.90 to 0.59) | -.11 | .72 (-0.70 to 0.49) |
| **8.2** Behavior substitution | -.22 | .09 (-0.48 to 0.04) | .006 | .06 (-0.18 to 0.20) | -.11 | .13 (-0.24 to 0.03) |
| **8.3** Habit formation | 0.10 | .90 (-1.42 to 1.61) | -.44 | .48 (-1.67 to 0.79) | -.02 | .97 (-0.94 to 0.90) |
| **8.4** Habit reversal | .13 | .89 (-1.76 to 2.02) | .17 | .81 (-1.23 to 1.58) | .49 | .36 (-0.57 to 1.54) |
| **8.5** Graded tasks | - | - | - | - | - | - |
| **8.6** Graded tasks | -.85 | .06 (-1.76 to 0.05) | .39 | .20 (-0.21 to 0.98) | -.28 | .21 (-0.71 to 0.16) |
| **9.1** Credible source | .23 | .75 (-1.27 to 1.72) | -.14 | .78 (-1.13 to 0.85) | .16 | .68 (-0.64 to 0.96) |
| **9.2** Pros and cons | -.69 | .05 (-1.39 to 0.01) | .28 | .19 (-0.14 to 0.71) | -.22 | .15 (-0.53 to 0.08) |
| **9.3** Comparative imagining of future outcomes | -.14 | .91 (-2.42 to 2.15) | -.37 | .68 (-2.13 to 1.40) | .05 | .94 (-1.18 to 1.38) |
| **10.1** Material incentive (behavior) | -.14 | .91 (-2.42 to 2.15) | -.37 | .68 (-2.13 to 1.40) | .05 | .94 (-1.18 to 1.38) |
| **10.2** Material reward (behavior) | - | - | - | - | - | - |
| **10.3** Non-specific reward | - | - | - | - | - | - |
| **10.4** Social reward (behavior) | - | - | - | - | - | - |
| **10.5** Social incentive | -.03 | .98 (-2.38 to 2.32) | .64 | .53 (-1.34 to 2.62) | .10 | .89 (-1.35 to 1.56) |
| **10.6** Non-specific incentive | - | - | - | - | - | - |
| **10.7** Self-incentive | -.44 | .26 (-1.23 to 0.35) | .007 | .98 (-0.56 to 0.57) | -.27 | .19 (-0.68 to 0.14) |
| **10.8** Incentive (outcome) | .60 | .70 (-2.55 to 3.74) | 1.12 | .37 (-1.20 to 3.24) | 1.14 | .17 (-0.47 to 2.74) |
| **10.9** Self-reward | .24 | .78 (-1.45 to 1.92) | -.37 | .55 (-1.58 to 0.84) | -.11 | .82 (-1.04 to 0.83) |
| **10.10** Reward (outcome) | - | - | - | - | - | - |
| **10.11** Future punishment | - | - | - | - | - | - |
| **11.1** Pharmacological support | - | - | - | - | - | - |
| **11.2** Reduce negative emotions | -.64 | .24 (-1.74 to 0.46) | .41 | .31 (-0.37 to 1.19) | .06 | .84 (-0.57 to 0.69) |
| **11.3** Conserving mental resources | - | - | - | - | - | - |
| **11.4** Paradoxical instructions | - | - | - | - | - | - |
| **12.1** Restructuring the physical environment | .004 | .98 (-0.32 to 0.33) | .01 | .93 (-0.23 to 0.25) | -.02 | .82 (-0.20 to 0.16) |
| **12.2** Restructuring the social environment | .18 | .54 (-0.42 to 0.78) | -.18 | .44 (-0.63 to 0.28) | -.24 | .15 (-0.58 to 0.09) |
| **12.3** Avoidance/reducing exposure to cues for the behavior | .11 | .51 (-0.22 to 0.43) | -.04 | .73 (-0.29 to 0.20) | .04 | .66 (-0.15 to 0.23) |
| **12.4** Distraction | -.19 | .11 (-0.42 to 0.04) | -.006 | .94 (-0.18 to 0.17) | -.005 | .94 (-0.14 to 0.13) |
| **12.5** Adding objects to the environment | -.18 | .42 (-0.64 to 0.28) | -.09 | .61 (-0.42 to 0.25) | -.04 | .78 (-0.29 to 0.22) |
| **13.1** Identification of self as a role model | .95 | .52 (-2.15 to 4.04) | .15 | .87 (-1.71 to 2.01) | -1.60 | .02 (-2.93 to -0.26)*^a^ |
| **13.2** Framing/reframing | -.24 | .14 (-0.56 to 0.08) | .09 | .44 (-0.14 to 0.32) | .03 | .75 (-0.16 to 0.22) |
| **13.3** Incompatible beliefs | - | - | - | - | - | - |
| **13.4** Valued self-identity | - | - | - | - | - | - |
| **13.5** Identity associated with changed behavior | -.18 | .55 (-0.80 to 0.43) | -.09 | 0.69 (-0.55 to 0.36) | .01 | 0.95 (-0.34 to 0.36) |
| **14.1** Behavior cost | - | - | - | - | - | - |
| **14.2** Punishment | - | - | - | - | - | - |
| **14.3** Remove reward | - | - | - | - | - | - |
| **14.4** Reward approximation | - | - | - | - | - | - |
| **14.5** Rewarding completion | - | - | - | - | - | - |
| **14.6** Situation-specific reward | - | - | - | - | - | - |
| **14.7** Reward incompatible behavior | - | - | - | - | - | - |
| **14.8** Reward alternative behavior | - | - | - | - | - | - |
| **14.9** Reduce reward frequency | - | - | - | - | - | - |
| **14.10** Remove punishment | - | - | - | - | - | - |
| **15.1** Verbal persuasion about capability | -.04 | .77 (-0.33 to 0.25) | .12 | .27 (-0.09 to 0.33) | .05 | .56 (-0.12 to 0.21) |
| **15.2** Mental rehearsal of successful performance | -.08 | .90 (-1.33 to 1.18) | .12 | .82 (-0.82 to 1.04) | .43 | .22 (-0.26 to 1.12) |
| **15.3** Focus on past success | .13 | .29 (-0.76 to 1.02) | -.23 | .51 (-0.91 to 0.45) | -.02 | .94 (-0.54 to 0.50) |
| **15.4** Self-talk | -.15 | .69 (-0.92 to 0.62) | -.21 | .47 (-0.78 to 0.36) | -.02 | .92 (-0.46 to 0.41) |
| **16.1** Imaginary punishment | - | - |  | - | - | - |
| **16.2** Imaginary reward | -.08 | .60 (-0.41 to 0.24) | .16 | .15 (-0.06 to 0.37) | .08 | .35 (-0.09 to 0.25) |
| **16.3** Vicarious consequences | - | - | - | - | - | - |

* p<.05 ^a^ Models would not converge due to low event or delivery rates

**Note:** A delivery score could not be computed for “11.1 Pharmacological support” as this BCT was delivered in all 149 consultations. “13.3 Incompatible beliefs is not included here as this BCT was not coded by any practitioner who had recorded at least 3 initial consultations.

**Supplementary material S3.**

Transcribed BCT examples based on 2012 NCSCT treatment guidance as delivered in primary care

**BM_10 EXPLAIN IMPORTANCE OF ABRUPT CESSATION** - Encourage the client to stop abruptly and explain why it is better to do so rather than cut down gradually.

**PRACTITIONER**: Just be aware, once you’ve stopped and you’ve done all this hard work – do not have one single cigarette – not, you know one can trigger you off and start you smoking again. You know you’re a non-smoker. Why would you buy any?

**Transcript 50, C158**

**PRACTITIONER**: If you stop – that’s it, you stop. Don’t trust yourself with one – well I’ll just try and see how I get on. Don’t, don’t even test yourself. Just say no that is it. Me finished with cigarettes.

**Transcript 64, C123**

**A5 GIVE OPTIONS FOR ADDITIONAL AND LATER SUPPORT** - Provide information to the client about options for additional support outside of the stop smoking programme and where these are available.

**PRACTITIONER**: Alright. You also get extra support with this because when you get your packet, there’s a number on the side and if you go to their webpage, which is MyLifeRewards.com. Put your number in, you will get a little bit of extra support if you want it.

**Transcript 73, C149**

**PRACTITIONER**: Oh well but it’s just if you wanted to, there’s a National Health Service Quit on there and it’s completely free, but it gives you tips on stopping smoking but also it texts you to say you how much you’re saving.

**Transcript 117, C117**

**PRACTITIONER**: Right, I’ll give you some things to read as well. You can look it up on the internet, there’s a bit of a support group on the internet I believe.

**Transcript 50, C70**

**RD2 EMPHASISE CHOICE** - Advise the client that they have to choose which stop smoking medication to use, but that you can help them to make an informed choice or advise the client to choose a quit date that works best around their upcoming commitments.

**PRACTITIONER**: I mean it’s completely up to you. I mean, like you said, you’ve tried single therapies on their own…whether you need two together…. like some of the patches will give you that background nicotine in your system. But some of us need to do something in the mouth and get that hit, like you would have a cigarette. Do you see what I mean?

**Transcript 142, C39**

**PRACTITIONER**: What sort of thing would you be more prone to liking do you think? Do you think it’s the act of smoking? Do you think that would help you or…. sucking a lozenge or chewing a bit of gum?

**Transcript 14, C191**

**PRACTITIONER**: So, you get to here and you will actually have to – you take day eight, you take day nine – you’re not enjoying your cigarettes. You do actually have to do everything with your head, where you say right, I’m stopping smoking tomorrow. You’ve actually sort of have to make that decision.

**Transcript 80, C94 - C95** *Discussing Champix medication*

**RI1 ASSESS CURRENT AND PAST SMOKING BEHAVIOUR** - Assess the amount of cigarettes smoked, age smoking was initiated, patterns of smoking behaviours.

**PRACTITIONER**: Is your smoking quite regular or are there certain things that trigger you to reach for a cigarette.

**Transcript 143, C24**

**PRACTITIONER**: Alright, so tell me how many do you smoke?

**Transcript 59, C22**

**PRACTITIONER**: How much are you smoking a day?

**Transcript 24 C18**

**RI2 ASSESS CURRENT READINESS AND ABILITY TO QUIT** - Ask the client if they are ready to stop smoking for good or ask the client if this is the right time to try to stop smoking.

**PRACTITIONER**: And how important is it for you give up smoking altogether at this attempt? Would you say it’s desperately important, very important, quite or not at all?

**Transcript 138, C31**

**PRACTITIONER**: Okay, that’s a good thing. And you’re really keen. You know you really want to do it?

**Transcript 41, C50**

**PRACTITIONER**: …do you feel it’s the right time to stop again?

**Transcript 45, C51**

**RI3 ASSESS PAST HISTORY OF QUIT ATTEMPTS** - Ask the client if they have ever quit smoking before, how long for.

**PRACTITIONER**: You said you’d stopped in the past. How did you stop before?

**Transcript 142, C18**

**PRACTITIONER**: So, tell me when did you stop smoking then? When have you had gaps in your smoking history?

**Transcript 104, C25**

**RI5 ASSESS NICOTINE DEPENDENCE** - Ask the client how soon after they wake up, they smoke a cigarette or ask the client how many cigarettes they smoke on average each day.

**PRACTITIONER**: …which cigarette would you find it the hardest to give up – would it be the first one?

**Transcript 130, C89**

**PRACTITIONER**: Um and…the other thing I was going to say…. how soon do you have a cigarette in the morning when you wake up?

**Transcript 34, C61**

**PRACTITIONER**: When you go to bed at night, do you smoke through the night?

**Transcript 12, C119**

**RI9 EXPLAIN HOW TOBACCO DEPENDENCE DEVELOPS** - Inform the client that repeated exposure to nicotine through smoking causes the brain to release dopamine, which can make smoking feel pleasurable.

**PRACTITIONER**: So… these are… the receptors in your brain… and this is a nicotine molecule and when you smoke, they go to the receptors, and they give off these little pleasurable feel-good factors if you like.

**Transcript 7, C127**

**PRACTITIONER**: Right, I’ve got it here. It’s the nicotine that makes you want to smoke, in as much as… this sort of explains, this is a picture of this sort of little goblet cell if you like in your brain and when you smoke, the nicotine goes into these little cells and the brains’ reaction is to release something called dopamine. And that makes you feel good.

**Transcript 113, C238, C240**

**RC1 BUILD GENERAL RAPPORT** - Establish a positive, friendly, and professional relationship with the client. This must be in regard to the behaviour (smoking/smoking cessation).

**PRACTITIONER**: So, you were telling me that you have cut down smoking… From 30… That’s amazing. Well done. And how long have you been doing that for?

**Transcript 8, C4, C6, C8**

**PRACTITIONER**: Fantastic. You’ve done the work already haven’t you? So, let’s have a look…

**Transcript 8, C139**

**RC3 EXPLAIN THE PURPOSE OF CO MONITORING** - Explain that cigarettes contain carbon monoxide, a poisonous gas that deprives the body of oxygen and that a simple breath test can be carried out to determine the client’s current CO levels.

**PRACTITIONER**: There’s also carbon monoxide. Now we can do a carbon monoxide read to show you how much carbon monoxide is in your…blood stream. Now carbon monoxide sits on the red blood cells where oxygen sits. So, it will tell us how much less oxygen you’ve got. As soon as you’ve stopped smoking, that will come back up.

**Transcript 7, C24**

**PRACTITIONER**: What I’d like to do next is to do a carbon monoxide reading. That just tells me to see how much carbon monoxide you’ve got running around in your blood stream. But also, it helps you because when you do it next time and you’re not smoking, you’ll have – it’s one where you can see how much cleaner your lungs have become.

**Transcript 143, C51**

**PRACTITIONER**: So, we’ll do a carbon monoxide blow. Not because it stops you smoking but when you see if it’s a high number and it reduces and it’s quite motivating and it makes feel like you’re actually achieving something.

**Transcript 1, C36**

**RC4 EXPLAIN EXPECTATIONS REGARDING THE TREATMENT PROGRAMME** - Inform the client that they are expected to have quit smoking within 14 days of the first stop smoking session.

**PRACTITIONER**: So, what happens now is I’ll give you a ring in a couple of weeks to see how you’re getting on. If there’s any problems obviously, we’ll have a chat about it and see what we can do to improve things. And then I need to see you again in about a month – you come back and blow into the machine and get another prescription.

**Transcript 115, C171**

**PRACTITIONER**: That’s what we do now because then we’d like to see you in a fortnight. If you need another prescription, we’ll get it. And if you don’t then we’ll change it.

**Transcript 142, C47**

**PRACTITIONER**: So long as you’re okay sort of by the middle of that week, you should be okay and be able to carry on taking it for the 12 weeks. So, from day 8 to day 14 – is where we suggest you pick a day to be your quit date.

**Transcript 10, C133**

**RC6 PROVIDE INFORMATION ON WITHDRAWAL SYMPTOMS** - Provide information to the client on the symptoms of nicotine withdrawal, including commonality, duration, and how to alleviate them.

**PRACTITIONER**: And a lot of people, when they stop, do get short time side effects. They feel irritable, dry mouth, a change of bowel habit, a change of appetite, sleeplessness, irritability.

**Transcript 116, C131**

**PRACTITIONER**: Giving up smoking with or without treatment causes various symptoms is – change of mood, feeling depressed, irritable, frustrated or anxious – sleeplessness, difficulty concentrating, okay…

**Transcript 100, C186**

**PRACTITIONER**: …you can get constipated, you can get flu like symptoms, greasy skin. You’ve got to remember all the stuff’s got to come out of the body somewhere that’s been put into it.

**Transcript 105, C152**

**RC7 USE REFLECTIVE LISTENING** - Provide agreement (e.g., verbal, non-verbal) on the client’s views (e.g., of stop smoking medications), summarising the discussion afterwards to confer understanding.

**PRACTITIONER**: Now you said – like you said in the morning…it could be first thing in the morning that you think about having another cigarette so we usually – we’d advise if you wanted – is the patches because it gives you that slow release…

**Transcript 112, C34**

**PRACTITIONER**: Okay so your kind of rewarding yourself for being really good and you’re rewarding yourself with cigarettes, aren’t you?

**Transcript 14, C139**

**RC8 ELICIT CLIENT VIEWS** - Ask the client their views on the stop smoking support received.

Prompt the client to express how they feel about the decisions that have been made (e.g., quit date, pharmacotherapy).

**PRACTITIONER**: Tomorrow – you’re not going out over the weekend or anything like that – that you’d be tempted? Okay, do you want to start tomorrow morning?

**Transcript 97, C179, C180**

**PRACTITIONER**: So, with regard to your quit date – so what are you feeling is a suitable time for you?

**Transcript 70, C84**

**PRACTITIONER**: So, is tomorrow a good day to quit?

**Transcript 12, C257**

**RC9 SUMMARISE INFORMATION/CONFIRM CLIENT DECISIONS** - Summarise what you expect the client to do before the next appointment (e.g., collect medication, quit) or confirm which medications the client has chosen.

**PRACTITIONER**: So, you get up tomorrow morning, you get that patch on. Use your inhalator; you’ve got 12 cartridges a day when you need it. You start to learn the knack…hold it in your mouth, let it go through. You keep remembering why you’re doing this. There’s lots of reasons you’re doing it. Your health, money – you haven’t got any! And you want to go and visit your nephews in Canada and you’re going to save for that and you’re going to look to that.

**Transcript 12, C259**

**PRACTITIONER**: So, we’ll go down the stairs in a minute and we’ll pick that prescription up and I’ll give you an appointment in one week’s time and…. that will be it, okay.

**Transcript 13, C122**

**1.1 GOAL SETTING (BEHAVIOUR)** - Agree on a quit date with the client

**PRACTITIONER:** So, we’re going to put a quit date down as the fourth, okay? So, we’ll put that down, so we’ve got the date to work towards.

**Transcript 27, C195**

**PRACTITIONER:** So, with regard to your quit date – so what are you feeling is a suitable time for you?

**Transcript 70, C84**

**1.2 PROBLEM SOLVING** - Identify specific triggers (e.g. being in a pub, feeling anxious) that generate the urge/want/need to smoke and develop strategies for avoiding environmental triggers that motivate smoking

**PRACTITIONER**: You need like a crossword or something. Try and think of things that you can do to replace that time to fill up boredom.

**Transcript 34, C47**

**PRACTITIONER**: They say that the addiction lasts…that the craving can last a few minutes, say it’s 10 minutes, okay. In that time, you can get up, go upstairs, clean your teeth, get all minty and fresh, come downstairs, make a cup of tea, walk round the garden, do a puzzle, read a book – do you see what I’m saying? Have lots of things around you.

**Transcript 12, C154**

**PRACTITIONER**: And it’s kind of getting out of that pattern of maybe just changing it just that little bit – do you understand – it’s changing that pattern of behaviour so you’re not kind of going along in that sequence as you would do normally.

**Transcript 33, C70**

**1.4 ACTION PLANNING** - Prompt planning of preparatory behaviour to facilitate quitting smoking (e.g. remove tobacco products from the house, tell others about the quit attempt) on specific days during the coming week or prompt the client to make a plan how, when and where medication will be used.

**PRACTITIONER**: So, I also advise people who come to see me – on the night before they’re giving up, or their quit date – get rid of everything to do with your cigarettes.

**Transcript 7, C155**

**PRACTITIONER**: You know once a week plan something – go to the cinema, let’s do something different so you know…it’s using that time differently.

**Transcript 135, C94**

**PRACTITIONER**: And then the morning you wake up, this is my quit date, get your inhalator ready. Pop it in your mouth if you want, put your patch on and off you go. Now if you normally shower in the morning – have your shower with the old patch on because it could come off.

**Transcript 1, C214**

**1.9 COMMITMENT** - Ask the person to use an “I will” statement to affirm or reaffirm a strong commitment (i.e. using the words “strongly”, “committed” or “high priority”) to quit smoking.

**PRACTITIONER**: Yeah, I mean are you really ready to do that? You really do want…. you’re really committed to stopping because it’s important that it’s what you want to do.

**Transcript 12, C35**

**2.2 FEEDBACK ON BEHAVIOUR** – Monitor and provide informative or evaluative feedback on performance of smoking/cessation *(e.g. form, frequency, duration, intensity)*

**PRACTITIONER**: Okay, so that’s a score of 2, 3, 4, 5, so you’ve got medium dependency

**Transcript 47, C142**

**PRACTITIONER**: 7. So you’re just at the high end of the medium dependency.

**Transcript 76, C92**

**2.6 BIOFEEDBACK** - Inform the person of their measured expired-air carbon monoxide concentration to encourage smoking cessation

**PRACTITIONER**: Okay so it’s 35, alright. It kind of goes red, amber, green. The red obviously being high. Amber then green but when I next see you – that will be below six, which will be normal, okay?

**Transcript 25, C57**

**PRACTITIONER**: Okay so that’s 18. Okay and that tells me that you smoke but we know that – you’ve already told me that, okay? But what you will notice is that once you stop smoking, it drops down to normal levels very, very quickly, which is good.

**Transcript 36, C66**

**3.1 SOCIAL SUPPORT (UNSPECIFIED)** - Advise the client to ask for social support during the quit attempt from family members, friends, or colleagues

**PRACTITIONER**: Support when you need it and that said, we can give you the support you need so we must see you regularly.

**Transcript 135, C117**

**PRACTITIONER**: Sometimes you just need that little bit of extra encouragement, don’t you, just to think no, we can do it, and we can hopefully help you with our support and…your determination…we’ll get there between us.

**Transcript 100, C61**

**PRACTITIONER**: Get them on board so they’re encouraging you and you know, just really behind you all the way because that is important because it is hard but it’s not impossible.

**Transcript 64, C91, C92**

**9.2 PROS AND CONS** – Advise the client to generate a list of reasons why they do and why they do not want to stop smoking

**PRACTITIONER**: This is like a little plan to stop smoking checklist. There’s various different things within here that you can fill out. Why you want to smoke, why do you want to stop to smoke. So, there’s the kind of pros and cons that you can lay out and always look back on. It’s always a good thing.

**Transcript 9, C70**

**11.1 PHARMALOGICAL SUPPORT** – Provide information, or encourage the use of or adherence to (including enacting necessary procedures to ensure that the client obtains medication easily), stop smoking medications to facilitate smoking cessation

**PRACTITIONER**: So, the way they work is you’re addicted to nicotine, so the nicotine doesn’t do you the harm. What we do is we give you a patch. It starts off at a strength and then we withdraw you slowly from the strength of nicotine, okay? Each course, whether it’s Champix or whether it’s a patch – is a 12-week course and the important thing is you must follow the course.

**Transcript 4, C63**

**PRACTITIONER**: So, you’ve got patches, you’ve got gum, you’ve got inhalators, you’ve got mouth sprays, all that sort of stuff. I find they’re okay for a top up but really, it’s easier just to get a patch on your arm and then you don’t think about it. If you struggle, we can then add one of the others to it, okay?

**Transcript 4, C62**

**PRACTITIONER**: All of them will wean you off the nicotine. Now there is also other medications erm like something called Champix which is a tablet. You take it while you’re still smoking, and it helps – it works on the receptors of your brain. Okay?

**Transcript 112, C18**

**12.1 RESTRUCTURING THE PHYSICAL ENVIRONMENT** - Advise the client to remove all tobacco products, lighters and ashtrays from their surroundings

**PRACTITIONER**: So, what I would recommend is that the night before, if you can, get rid of everything to do with your smoking – ashtrays, lighters, the lot.

**Transcript 1, C210**

**PRACTITIONER**: The last day of your smoking so next Wednesday, smoke as normal, get rid, when you go to bed – get rid of any cigarettes you’ve got left, any papers –

**Transcript 116, C226**

**12.3 AVOIDANCE/REDUCING EXPOSURE TO CUES FOR THE BEHAVIOUR** - Advise the client to avoid situations such as pubs in which common triggers to smoke occur by changing daily/weekly routines

**PRACTITIONER**: But you know, if you really really can’t control yourself when you go out – I’m not saying you can’t but if you really really feel you can’t say no at that point – a) you either stop going out for a couple of weeks until you’re over that point or you really just stay in the pub and say I’ll come out with you but we’ve got to sit inside the pub because I want to give up smoking.

**Transcript 35, C113**

**PRACTITIONER**: So, there’s associations you have with after a meal and smoking – you’re probably sitting down having a bit of time for yourself before you get busy again. It might mean that you need to go and do something else. Something relaxing but something that you can actually physically do so you’re not sitting there missing the cigarettes quite as much.

**Transcript 143, C40**

**13.5 IDENTITY ASSOCIATED WITH CHANGED BEHAVIOUR** - Ask the person to articulate their new identity as an ‘ex-smoker’

**PRACTITIONER**: From Wednesday morning, you are a non-smoker – if you tell yourself, your head enough times – I don’t smoke – and your body just sort…I mean it gets easier as you go on, as the nicotine receptors, you know – they’ve been – the little inhalator is the equivalent…

**Transcript 54, C162**

**PRACTITIONER**: If – once you’ve stopped, you’ve stopped smoking. That’s it, you’re a non-smoker. Why would you buy any cigs?

**Transcript 89, C154**

**15.1 VERBAL PERSUASION ABOUT CAPABILITY** - Tell the person that they can quit smoking even though previous quit attempts have not been successful

**PRACTITIONER**: I think people who have smoked a long time often maybe perceive that it’s going to be more difficult for them to give up but in actual fact you know it is possible for anybody, if they’ve got all the building blocks in the right place, the right support, the right motivation, the right frame of mind – can do.

**Transcript 116, C142**

**PRACTITIONER**: You do need the willpower behind it, which you’ve got. This is maybe just that little extra boost you need.

**Transcript 112, C55**

**15.3 FOCUS ON PAST SUCCESS** - Advise to think about or list previous successes in performing the behaviour (or parts of it)

**PRACTITIONER**: Yeah, I mean you’ve obviously done it before so remember what you did. Do not have any cigarettes in the house.

**Transcript 80, C144**

**PRACTITIONER**: You did for a year and now you can do it for another year then another year, then another year.

**Transcript 8, C28**

**PRACTITIONER**: Well, the thing is obviously… if the patches obviously have worked for you in the past before – it might sort of be worth going back down that route.

**Transcript 133, C68**
